# AMULET: a novel read count-based method for effective multiplet detection from single nucleus ATAC-seq data

**DOI:** 10.1186/s13059-021-02469-x

**Published:** 2021-09-01

**Authors:** Asa Thibodeau, Alper Eroglu, Christopher S. McGinnis, Nathan Lawlor, Djamel Nehar-Belaid, Romy Kursawe, Radu Marches, Daniel N. Conrad, George A. Kuchel, Zev J. Gartner, Jacques Banchereau, Michael L. Stitzel, A. Ercument Cicek, Duygu Ucar

**Affiliations:** 1 The Jackson Laboratory for Genomic Medicine, Farmington, CT 06032 USA; 2 Department of Pharmaceutical Chemistry, University of California, San Francisco, San Francisco, CA 94158 USA; 3 University of Connecticut Center on Aging, UConn Health Center, Farmington, CT 06030 USA; 4 Chan-Zuckerberg Biohub, San Francisco, CA 94158 USA; 5 NSF Center for Cellular Construction, San Francisco, CA 94158 USA; 6 Department of Genetics and Genome Sciences, University of Connecticut Health Center, Farmington, CT 06030 USA; 7 Institute for Systems Genomics, University of Connecticut Health Center, Farmington, CT 06030 USA; 8 Computer Engineering Department, Bilkent University, 06800 Ankara, Turkey; 9 Computational Biology Department, Carnegie Mellon University, Pittsburgh, PA 15213 USA

**Keywords:** Multiplets, Doublets, Single nucleus ATAC-seq, snATAC-seq

## Abstract

Detecting multiplets in single nucleus (sn)ATAC-seq data is challenging due to data sparsity and limited dynamic range. AMULET (ATAC-seq MULtiplet Estimation Tool) enumerates regions with greater than two uniquely aligned reads across the genome to effectively detect multiplets. We evaluate the method by generating snATAC-seq data in the human blood and pancreatic islet samples. AMULET has high precision, estimated via donor-based multiplexing, and high recall, estimated via simulated multiplets, compared to alternatives and identifies multiplets most effectively when a certain read depth of 25K median valid reads per nucleus is achieved.

**Supplementary Information:**

The online version contains supplementary material available at 10.1186/s13059-021-02469-x.

## Background

Single nucleus ATAC-seq (snATAC-seq) [1, 2] technology has accelerated the study of epigenetic regulation with single-cell resolution [3, 4]. However, the pace of computational method development for this assay lags behind the pace of data generation. An open computational problem is the detection of multiplets (i.e., two or more cells/nuclei captured and profiled together)—a common challenge for droplet-based single-cell technologies [5]. The presence of multiplets confounds downstream analyses, such as cell clustering, annotation, differential accessibility, and allelic accessibility analyses, by introducing combined epigenomic profiles that originate from two or more nuclei. Multiplet detection in snATAC-seq is a distinct computational challenge compared to single-cell RNA-seq assays due to data sparsity and the limited dynamic range of single-cell chromatin accessibility levels (e.g., 0 reads, closed chromatin; 1, open on one parental chromosome; and 2, open on both chromosomes).

Current state-of-the-art methods for multiplet detection in snATAC-seq data (i.e., SnapATAC [6] and ArchR [7]) are similar in nature to scRNA-seq multiplet detection methods (e.g., DoubletFinder [8] and Scrublet [9]). These methods simulate multiplets by combining genomic profiles of two or more distinct cells/nuclei present in the data to detect real multiplets based on their similarity to these simulated multiplets. They are therefore designed to detect multiplets originating from different cell types (i.e., heterotypic multiplets) and assume that genomic profiles of multiplets are unique and distinguishable from the genomic profiles of distinct cell types. Although reasonable, this assumption has important limitations that may lead to false positive or false negative annotations. For example, simulation-based methods may not detect multiplets of functionally similar cell types (e.g., naive and memory CD4^+^ T cells) since their genomic similarity is higher. In addition, these methods will not detect homotypic multiplets (i.e., originating from the same cell type), since their genomic profile resembles that of the underlying cell type. Filtering out homotypic multiplets is important for the accuracy of certain downstream analyses (e.g., allelic bias and lineage tracing). To overcome these limitations and leverage inherent data features of snATAC-seq maps, we developed a novel computational framework, AMULET (**A**TAC-seq **MUL**tiplet **E**stimation **T**ool), that effectively captures and annotates multiplets by taking advantage of expected read count distributions per genomic region for each nucleus.

To benchmark the performance of AMULET, we generated snATAC-seq data from human peripheral blood mononuclear cells (PBMCs) (*n*=5 donors, 2 captured and sequenced individually, 3 pooled and sequenced together) and pancreatic islets (*n*=2 donors, captured and sequenced individually). AMULET’s efficacy was quantified based on its ability to detect and annotate artificially introduced multiplets and to detect known multiplets within multiplexed donor profiles. For sufficiently sequenced samples (i.e., > 25k valid read pairs per nucleus), AMULET detected heterotypic multiplets with high recall (0.85 in PBMCs), significantly outperforming simulation-based method ArchR (recall=0.24). With lower read counts per nucleus, ArchR outperformed AMULET (average recall 0.49 versus 0.36 in islets), but the two algorithms uncovered complementary sets of multiplets. Unlike simulation-based methods, AMULET was equally effective at detecting homotypic and heterotypic multiplets (recall=0.87 for PBMCs, 0.35 for islets). AMULET predictions were also higher in precision compared to ArchR (0.61 vs. 0.28) for the detection of multiplets from 3 multiplexed PBMC donors identified via genotype-based demultiplexing [10]. Finally, to gain further insights into the types and cellular origins of multiplets, we also developed a novel and effective clustering-based algorithm to annotate them. AMULET (https://ucarlab.github.io/AMULET/) is a user-friendly computational framework that can effectively detect and annotate multiplets in snATAC-seq and can be easily integrated into existing computational pipelines such as Signac [11], Seurat [12, 13], and ArchR [7].

## Results

**Fig. 1:**
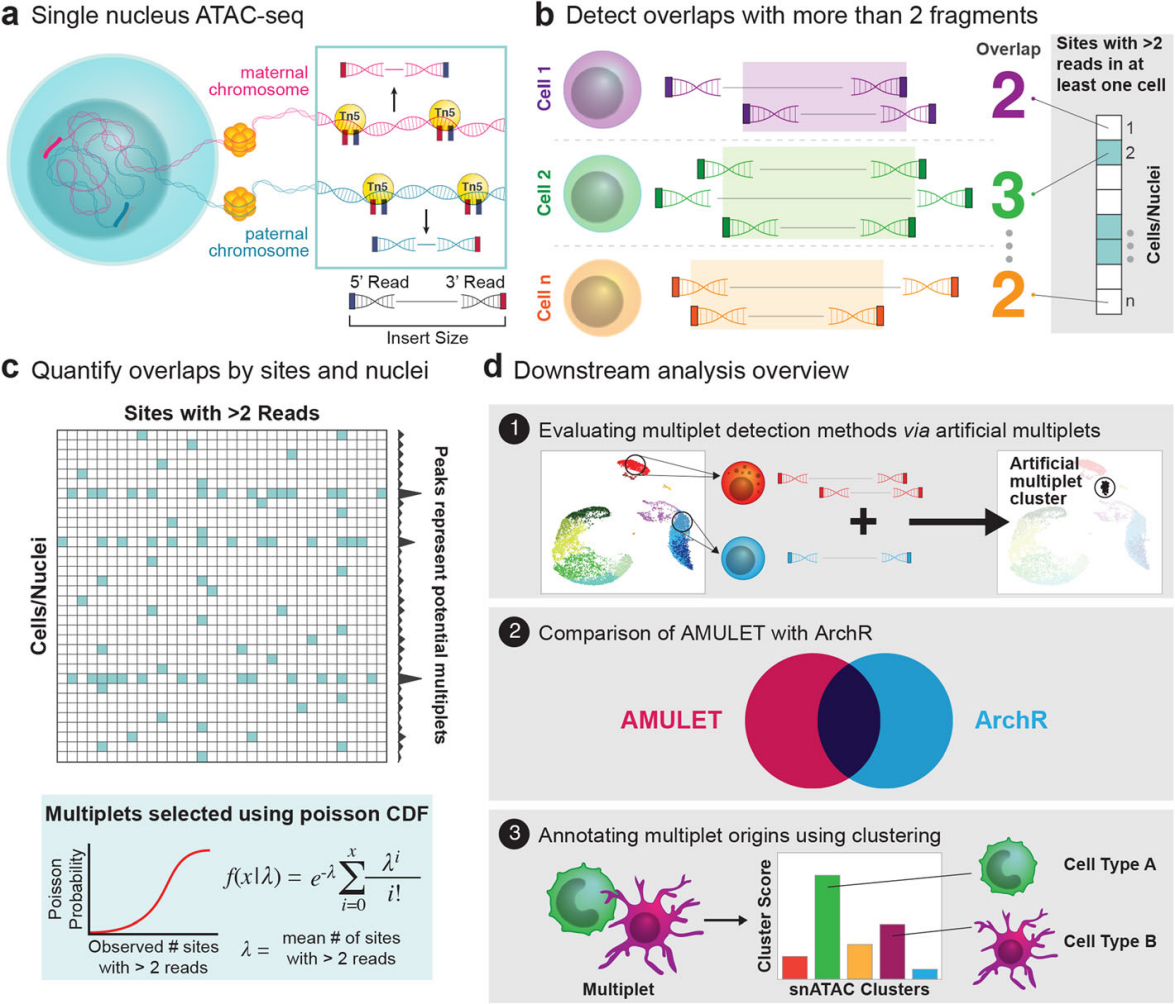
Overview of AMULET. **a** Tn5 transposase cleaves accessible DNA at maternal and paternal chromosomes in each nucleus. Number of ATAC-seq read counts per genomic region per nucleus are expected to be 0, 1, or 2. **b** Instances where >2 overlapping reads are observed for any genomic region in a cell are identified using an efficient algorithm for counting the number of overlapping reads. **c** Poisson cumulative distribution function is used to detect multiplets based on deviations from expected number of genomic regions with >2 overlapping reads. **d** Overview of downstream analyses: (1) quantification of multiplet detection performances using artificial multiplets, (2) comparison of AMULET to alternative method ArchR, and (3) annotating cellular origins of multiplets using a clustering-based method

AMULET leverages the principle that the expected number of uniquely aligned reads overlapping any given open chromatin region ranges from 0 to 2 for diploid nuclei in snATAC-seq data: 0 = closed chromatin, 1 = open on one chromosome (i.e., either the maternal or the paternal chromosome), and 2 = open on both chromosomes (i.e., both parental chromosomes) (Fig. 1a). In this assay, more than two overlapping reads (>2) can align to a genomic region due to (1) repetitive sequence content, (2) PCR duplication/jackpot effects, (3) sequencing/alignment errors, or (4) capture of multiple nuclei in one droplet (multiplets). The type of overlapping reads resulting from repetitive element sequences or experimental/technical errors will be localized to specific sites that can be filtered out using known repetitive elements and will be less frequent across the genome. In contrast, the capture of multiplets will yield a systematic, genome-wide increase in regions with >2 overlapping reads.

AMULET first identifies all sites with >2 reads for each nucleus (Fig. 1b) by utilizing sorted read alignments to detect those with overlapping read intervals. A unified list of these regions across all nuclei is generated and filtered using known repetitive elements (Methods) to quantify the number of occurrences where >2 reads align to a region in a given nucleus (Fig. 1c). Next, it models random occurrences of regions with >2 reads (i.e., due to experimental or sequencing/alignment errors) with the Poisson cumulative distribution function. Based on their deviations from the observed Poisson distribution using false discovery rate (FDR), nuclei determined to contain significantly more regions with >2 reads are identified as multiplets (Fig. 1c, an example shown in Additional file 1: Figure S1).

Detected multiplets are assigned to their cell type(s) of origin using a clustering-based algorithm within the AMULET framework. First, marker peaks for each cell type are detected via differential analyses in snATAC-seq data. For each nucleus, epigenomic similarity profiles are calculated by studying read count distributions at cell-type-specific marker peaks. These profiles are then used to trace back the cellular origins of multiplets and differentiate between heterotypic and homotypic multiplets. Performance of AMULET has been compared to simulation-based alternative ArchR [7] by generating reference snATAC-seq data in two primary human tissues (Fig. 1d).

### PBMC and islet snATAC-seq profiling to benchmark AMULET

**Fig. 2:**
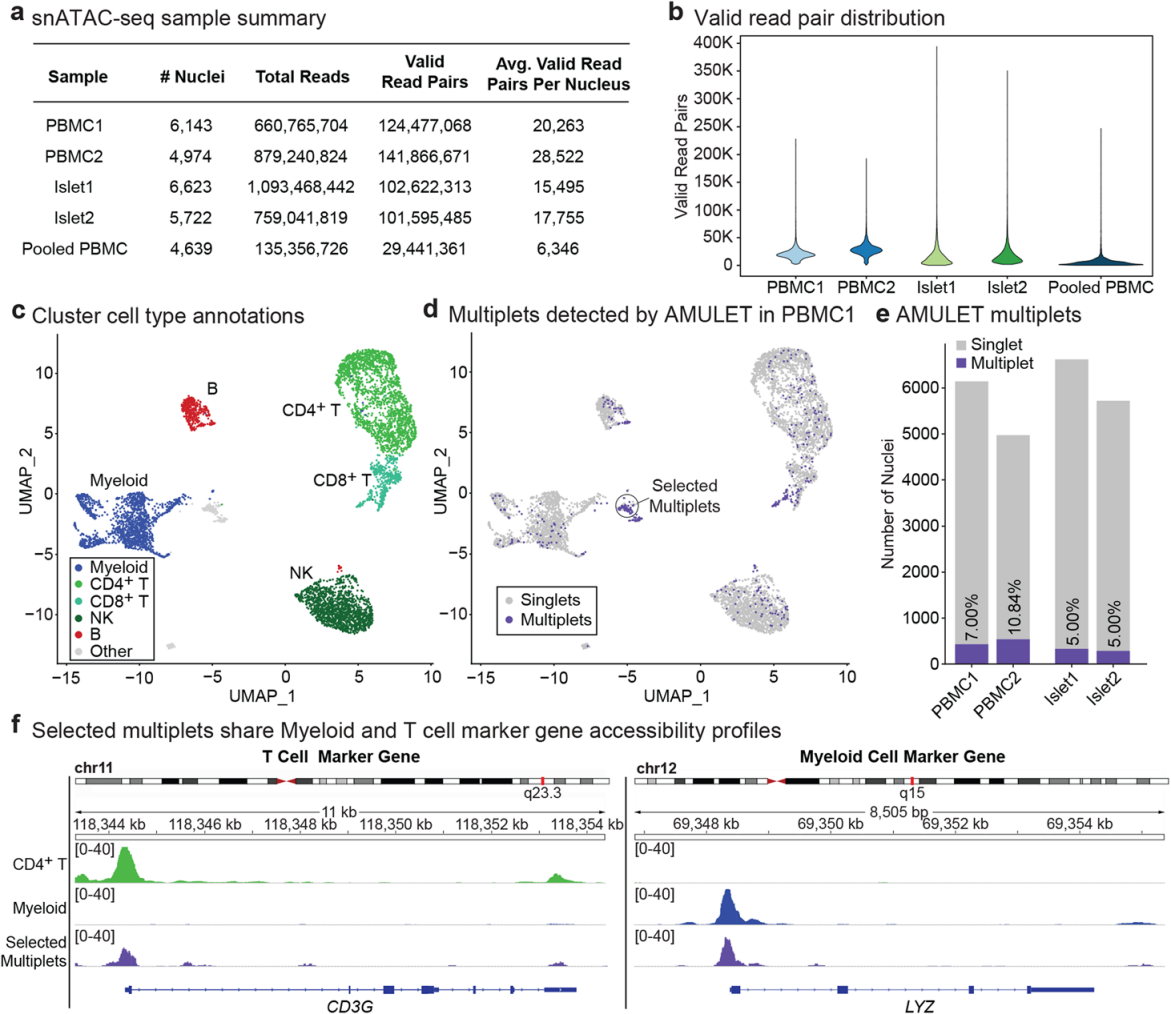
AMULET identifies heterotypic and homotypic snATAC-seq multiplets in primary human tissues. **a** Summary of snATAC-seq samples generated and used in this study from human PBMCs and islets. **b** Valid read pair distributions for PBMC and islet snATAC-seq samples. **c** PBMC clusters were annotated based on their correlations with sorted bulk ATAC-seq data. **d** All multiplets (heterotypic and homotypic) detected by AMULET in PBMC1. Selected multiplets refer to multiplets for which aggregated profiles are shown in panel **f** of this figure. **e** The number of cells and percentage of multiplets detected by AMULET in PBMC and islet samples. **f** Chromatin accessibility profiles of CD4^+^ T, myeloid, and selected multiplets around for T cell marker gene (*CD3G*) and myeloid cell marker gene (*LYZ*). CD4^+^ T and myeloid cells show strong accessibility signals for their relevant marker genes while selected multiplets have accessible chromatin for both marker genes

To benchmark AMULET’s performance, we generated snATAC-seq data in two primary human tissues: peripheral blood mononuclear cells (PBMCs) (*n*=5 donors: 2 donors independently captured and sequenced (PBMC1, PBMC2) and 3 donors that were pooled and sequenced together) and islets (*n*=2 donors, islet 1 and islet 2) using the 10x Genomics Chromium platform [3]. Sequence reads were pre-processed using the Cell Ranger ATAC pipeline (Methods), yielding an average of 5559 and 6173 nuclei per sample and 24,393 and 16,625 valid read pairs per nucleus for independently sequenced PBMC and islet samples, respectively (Fig. 2a). In this context, a valid read pair is a paired-end sequence aligning to an autosomal chromosome and passing quality control flags/thresholds (Methods). Despite deeper sequencing for islet samples, fewer valid read pairs per nucleus were observed in islet samples compared to PBMCs (Fig. 2b). This is likely due, at least in part, to the increased representation of mitochondrial reads in islet samples (115M and 48M chrM reads in islet 1 and islet 2, respectively) compared to PBMCs (2.6M and 0.95M chrM reads in PBMC1 and PBMC2).

Nuclei were clustered using a two-pass clustering method [3] (Methods) resulting in 16 and 15 clusters for PBMC1 and PBMC2. By correlating pseudo-bulk accessibility profiles of these clusters with accessibility maps from sorted bulk ATAC-seq data [14] (Additional file 1: Figure S2a,b), we annotated 5 major cell types: myeloid (including CD14^+^, CD16^+^ monocytes, and conventional dendritic cells), B, CD4^+^ T, CD8^+^ T, and NK cells (Additional file 1: Figure S2c,d). These annotations were confirmed based on the chromatin accessibility patterns at literature-supported marker genes (Additional file 1: Figure S3). The same clustering workflow identified 14 and 12 clusters for islet 1 and islet 2, which we annotated as alpha, beta, delta, and ductal cells, the most frequent cell types in islets, based on known marker genes [15] (Additional file 1: Figure S4).

After cell type annotation, we applied AMULET to detect high confidence (FDR < 0.01; Methods) multiplets in the PBMC and islet snATAC-seq datasets. Predicted multiplets were distributed within each cell-type cluster in all four samples (Fig. 2c, d, Additional file 1: Figure S5). In PBMC1, multiplets also formed their own distinct cluster (cluster 13 in Additional file 1: Figure S2c) (see selected multiplets in Fig. 2d). The percentage of detected multiplets was higher in PBMCs (7%, 10.84%) compared to islets (5% for both samples) (Fig. 2e), likely due to the higher number of valid read pairs per nucleus in PBMCs compared to islets (Fig. 2b). To determine the accuracy of AMULET multiplet predictions, we analyzed chromatin accessibility profiles (Fig. 2f) of a cluster exclusively comprised of multiplets in PBMC1 (Fig. 2d). These multiplets were characterized by high chromatin accessibility at the promoters of both *CD3G* (T cell marker gene) and *LYZ* (monocyte marker gene), confirming the T cell monocyte composition of these multiplets and supporting the utility of AMULET for snATAC-seq doublet detection.

### AMULET detects multiplets with high precision and recall

To quantify the efficacy of AMULET, we introduced artificial multiplets by randomly selecting 5% of nuclei in each dataset and forming nuclei pairs by adding their read count profiles together (repeated 10 times per sample). This random selection was independently done to simulate heterotypic and homotypic multiplets by taking cell type annotations into consideration. This generated artificial multiplets that constitute 2.5% of all nuclei in the sample to serve as true multiplet examples, enabling us to measure recall (i.e., the fraction of detected artificial multiplets to all artificial multiplets introduced in the sample). Using the same artificial multiplets for each comparison, we quantified AMULET and ArchR’s ability to detect both heterotypic and homotypic multiplet types.

**Fig. 3:**
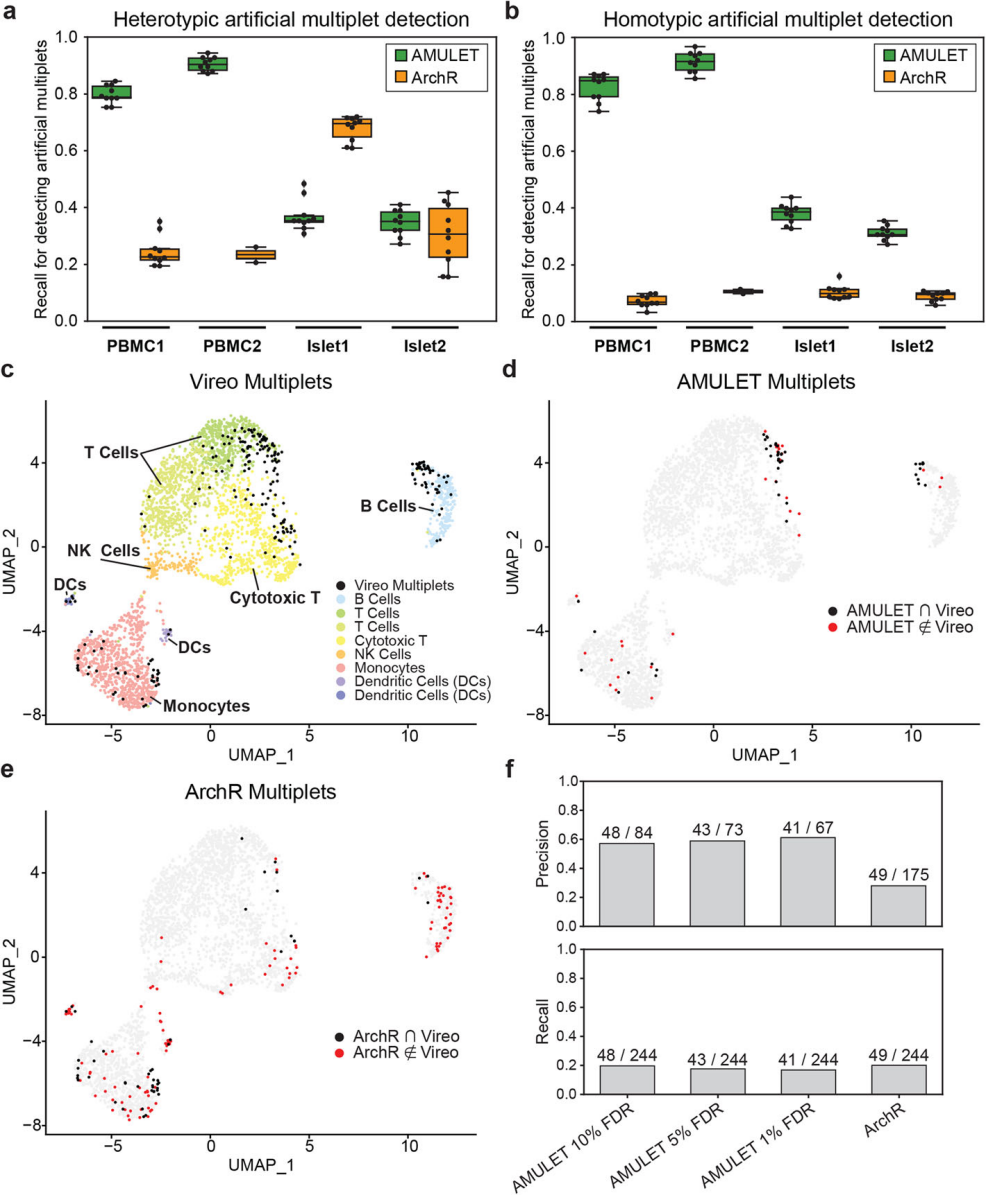
AMULET detects multiplets with high precision and recall. **a**, **b** Recall for detecting **a** heterotypic and **b** homotypic artificial multiplets. AMULET consistently detected both heterotypic and homotypic multiplets with similar recall, while ArchR was only effective for predicting heterotypic multiplets for data with high heterogeneity. **c** Unsupervised clustering of multiplexed PBMC data identified 8 clusters corresponding to B cells, T cells, cytotoxic T, NK cells, monocytes, and dendritic cells. Multiplet detection via Vireo identified 244 multiplets among donors. **d** Multiplets identified by AMULET (red) and both AMULET and Vireo (black). 59% of AMULET multiplets overlapped with Vireo multiplets, showing high precision for detecting known multiplets among donors. **e** Multiplets identified by ArchR (red) and both ArchR and Vireo (black). Only 28% of multiplets detected by ArchR were also multiplets detected by Vireo. **f** Comparison of precision and recall for AMULET (FDR 10%, 5%, and 1%) and ArchR (default parameters). AMULET has higher precision than ArchR (0.57–0.61 vs. 0.28) for all FDR cutoffs with similar recall (0.17–0.20 vs. 0.20)

In PBMC samples, AMULET had a high recall for detecting heterotypic multiplets (average recall 0.80 for PBMC1 and 0.90 for PBMC2 over 10 runs), substantially outperforming ArchR (0.25 and 0.23, respectively) (Fig. 3a). However, for islet 1 and islet 2, where the number of valid read pairs per nucleus was lower, average recall for AMULET was reduced to 0.37 and 0.35, respectively, while the average recall for ArchR predictions were 0.68 and 0.30, respectively. AMULET was similarly effective in detecting homotypic multiplets (average recall 0.82 and 0.91 for PBMC 1 and PBMC 2, 0.38 and 0.31 for islet 1 and islet 2) (Fig. 3b), whereas ArchR mostly missed these multiplets (average recall ranges from 0.07 to 0.11 for all samples) since homotypic multiplet epigenomic profiles are indistinguishable from bona fide singlets.

High recall may result from many false positive multiplet calls. Therefore, it is also critical to assess precision, which quantifies the number of positive class predictions that actually belong to the positive class (i.e., the ratio of true multiplets detected by the method). However, calculating precision is challenging without ground-truth singlet and multiplet classifications. Here, we estimated the lower bound for precision by assuming the worst-case scenario; i.e., all nuclei that are called as multiplets and not simulated as one are false-positive calls. Accordingly, even in the worst-case scenario AMULET had a higher average precision than ArchR for heterotypic (0.23 versus 0.06 for PBMCs, 0.16 versus 0.11 for islets) and homotypic (0.25 versus 0.02 for PBMCs, 0.16 versus 0.02 for Islets) cases (Additional file 1: Figure S6). Note that the best-case scenario for precision assumes that all detected multiplets are true positives; hence, the upper bound for precision for both methods and for all datasets will be 1 (i.e., perfect precision).

To better estimate the precision of multiplet calls, we generated multiplexed snATAC-seq data from the PBMCs of 3 individuals that were pooled and sequenced together. These samples were demultiplexed using Vireo [10] based on naturally occurring genetic variation in each individual, which also effectively detects multiplets among donors. Although multiplets among donors are a subset of all true multiplets, these data still enable an unbiased framework to benchmark different methods in terms of their precision. By using Vireo, we detected 244 true multiplets formed from the nuclei of different donors out of 3812 nuclei (Fig. 3c), providing an unbiased ground truth to estimate precision for donor multiplets. We then applied AMULET (FDR 1%, 5%, 10%) and ArchR (default parameters) to these data to detect 67–84 and 175 multiplets, respectively. 59% of AMULET multiplets (at 5% FDR) were also Vireo multiplets (Fig. 3d), while only 28% of ArchR multiplets were also identified by Vireo (Fig. 3e). Accordingly, AMULET detected Vireo multiplets with higher precision (0.57–0.61) compared to ArchR (0.28); both methods achieved a similar recall, 0.17–0.20 and 0.20, respectively (Fig. 3f).

Together, these results suggest that read-count-based AMULET can detect multiplets with high precision (lower false positives assessed by sample multiplexing) and high recall (lower false negatives assessed by simulated multiplets), especially when samples are sequenced deeply (e.g., 20–28K average, 19–28K median valid read pairs for PBMC1 and PBMC2), serving as an effective alternative to simulation-based methods.

### Multiplets detected in PBMCs and islets with AMULET and ArchR

**Fig. 4:**
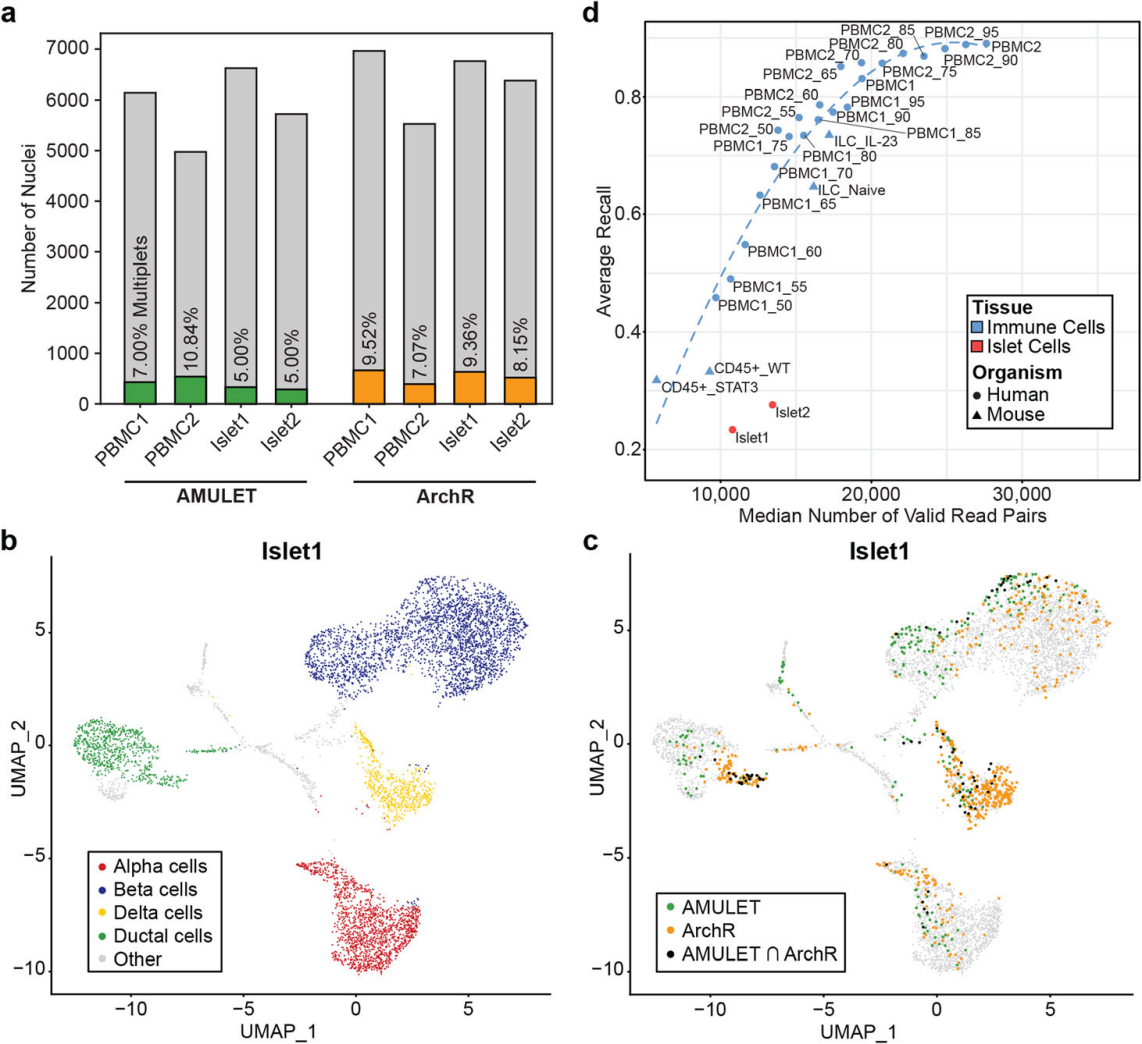
AMULET is robust to cell type similarity and improves increased with read depth. **a** Total number of nuclei and multiplets detected by each method. Differences in number of nuclei are due to differences in inputs (i.e., alignment (BAM) files and Cell Ranger cell annotations for AMULET, fragment files (Cell Ranger output) and ArchR cell annotations for ArchR). Overall, ArchR detects more nuclei as multiplets using default parameters than AMULET. **b** Reference annotations for islet 1. Islet 1 annotations correspond to alpha, beta, delta, and ductal cell types. **c** Multiplets detected by AMULET and ArchR for islet 1. Majority of multiplets detected were not shared between the two methods. Note: ArchR detected the majority of delta cells as multiplets. **d** Average recall for detecting artificially introduced multiplets in immune and islet samples with respect to median read count per nucleus. AMULET reaches its peak performance for ~25K median valid read pairs per nucleus. Differences between immune and islet cells likely stem from the overall differences in their accessibility levels and patterns

After benchmarking the two methods, we applied AMULET and ArchR on PBMCs and islets to detect multiplets in these samples. Each algorithm detected similar percentages of multiplets in both tissues: 5–11% with AMULET and 7–10% with ArchR (Fig. 4a). Multiplets detected by both methods were located on the periphery of single-cell type clusters or formed their own distinct clusters (Fig. 4b, c, Additional file 1: Figure S7a-c) comprised of mostly heterotypic multiplets with unique epigenomic profiles, which are more readily detected by simulation-based methods. However, their outputs were mostly disjointed (Additional file 1: Figure S7d). For example, in PBMC1, ArchR detected 663 multiplets compared to 430 multiplets from AMULET, of which only 75 overlapped. In islet profiles, delta cells were disproportionately labeled as multiplets using ArchR (47% in islet 1, Additional file 1: Figure S8a), likely due to their epigenomic similarity to beta cells (Additional file 1: Figure S8b). In contrast, AMULET multiplets were more balanced across cell types.

### AMULET’s performance improves with increased library depth

AMULET’s recall was better in highly sequenced PBMC samples compared to islets (Fig. 3a, b). To further study the library depth effects on AMULET’s performance, we applied it on snATAC-seq data with different library depth: (1) publicly available mouse CD45^+^ cells [16] and innate lymphoid cells (ILCs) data [17] and (2) down-sampled PBMC1 and PBMC2 samples at 5% increments starting at 50%. To quantify AMULET’s performance, we simulated multiplets in each sample by randomly selecting nuclei pairs and used them to calculate recall. For immune cells (PBMCs, CD45^+^ cells, and ILCs), AMULET achieved 88–89% recall for samples when the median read count per nuclei was 25–28K valid read pairs, and we observed a linear trend between recall and read count per nuclei until a certain depth is achieved (Fig. 4d). The variation in AMULET’s performance between immune and islet cells is likely due to the differences in their overall chromatin accessibility levels and patterns that might arise from distinct cellular differentiation potential and/or functions. We further investigated the number of valid read pairs required to detect multiplets using AMULET by randomly pairing 5% of the total nuclei per sample (repeated 100 times). We used AMULET to detect these artificial multiplets, then studied the read count distribution of the ones that are accurately identified. These analyses revealed that in both PBMCs and islets, ~25K valid read pairs per cell/nucleus is sufficient for detecting artificial multiplets with high recall using AMULET (Additional file 1: Figure S9). Together, these analyses show that AMULET’s performance depends on the read depth and that maximum performance is achieved when the median read count per nuclei is at least 25K valid read pairs regardless of the tissue type.

### The likelihood of a cell type to form multiplets is associated with its frequency within the tissue

**Fig. 5:**
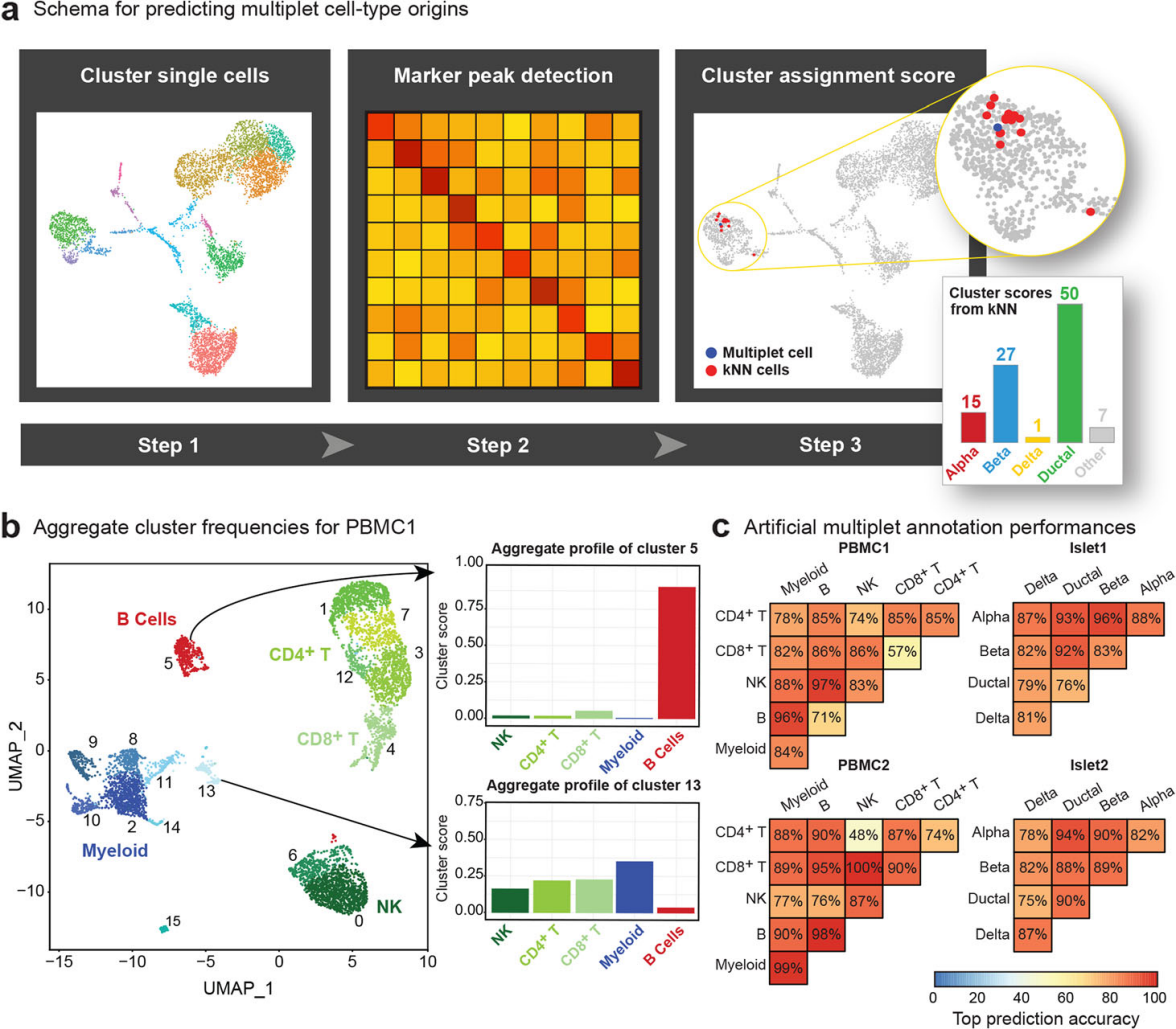
Multiplet cell-type origins are predicted with high accuracy. **a** Overview of the cell origin annotation pipeline. First, cells are clustered. Second, marker peaks are identified. Third, multiplets and their k-nearest neighbor cells are used to generate cluster similarity scores. **b** Example of aggregate cluster profiles for predicting cell origin annotations. Clusters corresponding to cell types observe strong signal for their respective cell types (e.g., cluster 5) while clusters corresponding to multiplets show a mixed profile of cell types (e.g., cluster 13). **c** Heatmaps of cell origin annotation accuracies for predicting artificial multiplets derived from cells of the specific cell type pairings. Multiplet annotations showed high accuracies for the majority of cell type compositions

To understand whether certain cell types are more likely to form multiplets and to gain insights into the types (heterotypic versus homotypic) and potential sources of multiplets in snATAC-seq data, we developed a multiplet annotation method (Fig. 5a). For this, we first identify marker peaks for each cell type and then calculate each nuclei’s likelihood to belong to these cell types using their epigenomic signal at marker peaks (cell type association score, Methods). For example, in PBMCs, we calculated five scores for each nucleus, which correspond to the five major cell types studied here (Fig. 5b). As expected, nuclei in cluster 5 (B cell cluster) had high scores for B cell marker peaks, whereas nuclei in cluster 13 (i.e., multiplet cluster) had high scores for multiple cell types: NK, CD4^+^ T, CD8^+^ T, and myeloid cells (Fig. 5b). With this framework, we can infer the cellular origins of each multiplet and also distinguish the type of the multiplet (heterotypic versus homotypic) (Methods).

First, we evaluated the efficacy of this multiplet annotation pipeline using artificial multiplets. Our method inferred the cellular origins of artificially simulated homotypic and heterotypic multiplets with high accuracy: 82% and 86% in PBMC1 and PBMC2, and 86% and 85% in islet 1 and islet 2 (Fig. 5c). For example, in PBMC1, 96% of all simulated B and myeloid multiplets were correctly annotated, whereas annotations of artificial multiplets generated from cells with similar functions were less accurate (e.g., 86% for simulated NK and CD8^+^ T cell multiplets), since they have similar epigenomes. Our framework was similarly effective for annotating homotypic and heterotypic multiplets, showing 84% accuracy on average to annotate homotypic multiplets and 86% accuracy to annotate heterotypic multiplets in both tissues.

**Fig. 6:**
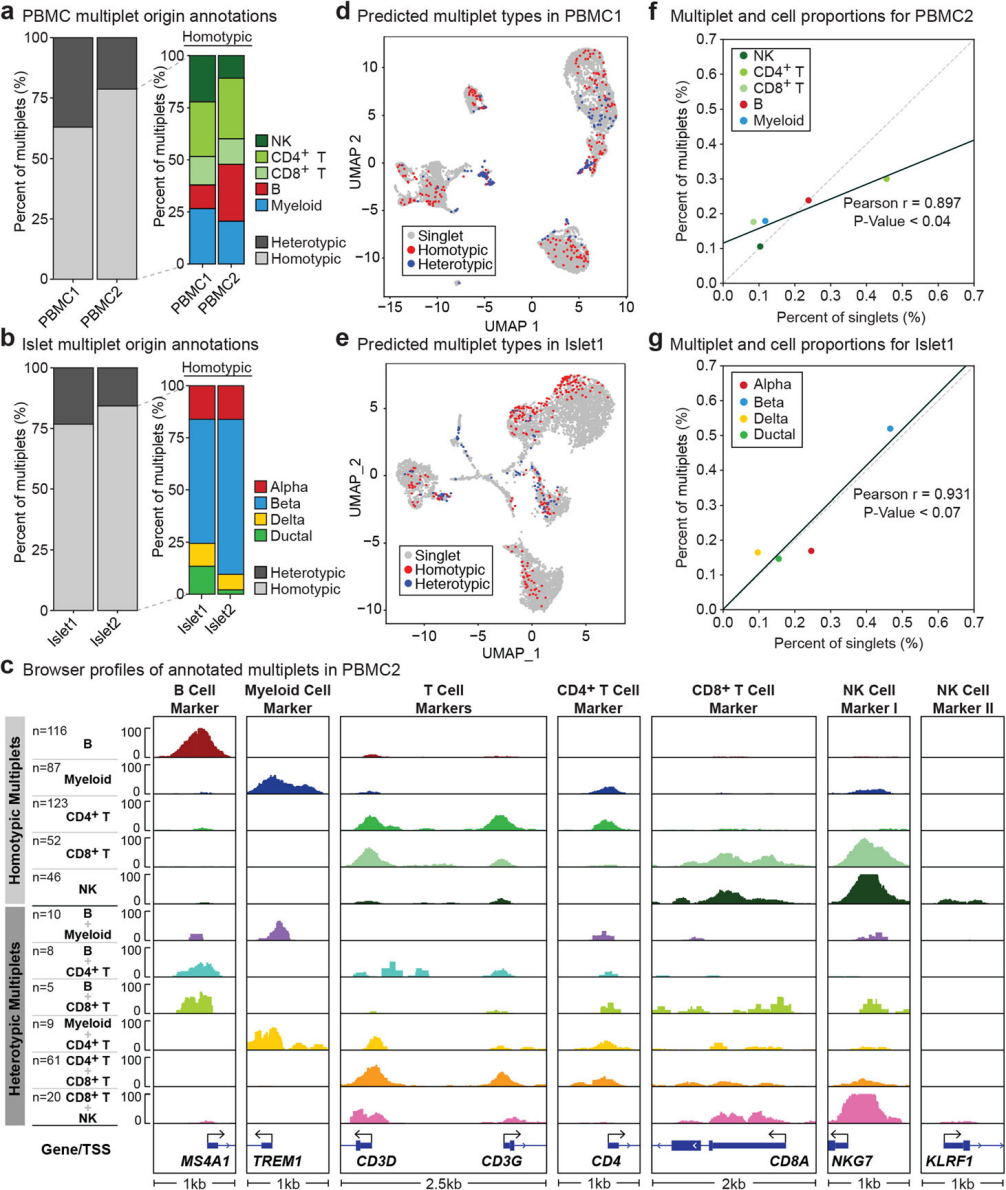
Majority of multiplets are homotypic and correspond to cell type proportions. **a**, **b** Heterotypic and homotypic multiplet cell distributions (left bars). Homotypic cell type annotations (right bars) for PBMC (**a**) and islet (**b**) samples. Majority of multiplets are annotated as homotypic. Homotypic cell type distributions show similar distribution to the overall proportions of each cell type in their respective samples. **c** Accessibility maps for cell origin annotations for multiplets identified in PBMC2. Homotypic multiplets observe strong signal for their respective marker genes. Heterotypic multiplets observe a combined signal at respective marker genes corresponding to the respective annotated cell types. **d**, **e** UMAP clustering for heterotypic and homotypic multiplet annotations in PBMC1 (**d**) and islet 1 (**e**). Heterotypic multiplets are found between major cell type clusters. Homotypic multiplets are observed on the periphery of major cell type clusters. **f**, **g** Cell and multiplet proportions for PBMC2 (**f**) and islet 1 (**g**). Multiplet cell type proportions are highly correlated with overall cell proportions

Next, we applied this annotation framework on multiplets detected from PBMCs and islets, which revealed that the majority of multiplets are homotypic: 77–84% in islets, 63–79% in PBMCs (Fig. 6a, b, Additional file 1: Figure S10a,b). Chromatin accessibility profiles at marker gene promoters for each cell type confirmed that homotypic and heterotypic multiplets have distinct profiles. For instance, homotypic B cell multiplets had strong accessibility signal for B cell marker gene *MS4A1* and not for marker genes of other cell types, whereas heterotypic multiplets originating from CD8^+^ T cell and B cells had high accessibility signals for both B cell marker gene *MS4A1* and CD8^+^ T cell marker gene *CD8A* (Fig. 6c for PBMC2). In both tissues, homotypic multiplets clustered together with the underlying cell type, whereas heterotypic multiplets either formed their own clusters or were located between cell type clusters (Fig. 6d, e, Additional file 1: Figure S10c,d). Notably, the delta cell cluster in islet 1 also included many heterotypic multiplets, supporting the hypothesis that these cells are similar to multiple major cell types in islets and are more likely to be mistaken as multiplets by simulation-based multiplet detection methods.

Further inspection of multiplet annotations revealed that the likelihood of a cell type to form a multiplet is positively correlated with the proportion of that cell type within the tissue (Pearson’s *R* = 0.824, 0.897, *P* values < 0.087, 0.04 for PBMC1 and PBMC2, Pearson’s *R* = 0.931, 0.475 *P* values < 0.07, 0.525 for islet 1 and islet 2) (Fig. 6f, g, Additional file 1: Figure S10e,f), suggesting that snATAC-seq multiplets are more likely to be technical (i.e., due to random coupling of nuclei) than biological (e.g., due to cellular interactions). For example, the most abundant cell type in islet 1 was beta cells (46.62% of the cell population) which contributed to 51.96% of multiplets (Fig. 6g). Heterotypic multiplet annotations in islet samples mostly originated from alpha, beta, and delta cells (Additional file 1: Figure S10b). In PBMCs, the most frequent heterotypic multiplets contained CD4^+^ T or CD8^+^ T cells (Additional file 1: Figure S10a).

## Discussion

Detecting and removing multiplets from snATAC-seq data is an important step to improve the quality and accuracy of downstream analyses. AMULET exploits unique data features of snATAC-seq assays to detect and eliminate multiplets, offering a read count-based alternative to current simulation-based methods (e.g., the method implemented as part of the ArchR framework). This read count-based approach equips AMULET to uniquely and effectively detect both heterotypic (i.e., multiplets originating from different cell types) and homotypic (i.e., multiplets originating from the same cell type) multiplets. Eliminating heterotypic multiplets is essential for improved clustering and differential analyses between clusters and samples, whereas eliminating homotypic multiplets can improve allele-specific or lineage tracing analyses that require accurate read counts per nucleus. AMULET was designed to detect multiplets in diploid cells, by systematically searching for sites with >2 read counts. This method can be improved in the future to analyze snATAC-seq data from haploid cells (e.g., gametes), polyploid cells, or aneuploid cancer cells by adjusting the expected number of reads per genomic region parameter or restricting the analyses to specific portions of the genome.

AMULET detected multiplets with high precision (assessed by sample multiplexing) and high recall (assessed by simulated multiplets), especially when samples are sequenced to a certain read depth, serving as an effective alternative to simulation-based ArchR. In depth analyses of published data from other groups and data from our studies showed that AMULET reaches its maximum performance (recall is ~90% for immune cells) when on the average each nucleus has ~25K valid read pairs (i.e., uniquely aligning, autosomal read pairs). In both tissues, this is achieved when a sample has ~120–130K total reads (prior to any data filtering and quality control) per nucleus on the average. Since AMULET does not depend on simulated multiplets, it is equally effective for cell types that are functionally similar, and hence share similar transcriptional regulatory architecture. For example, in islets, delta cell epigenomes resemble that of beta cells (Additional file 1: Figure S8b). These instances are particularly challenging for simulation-based methods (e.g., ArchR for snATAC [7] or DoubletFinder [8] and Scrublet [9] for scRNA-seq) as evident by the fact that ArchR categorized 47% of delta cells as multiplets in islet 1 while AMULET categorized just 11% as multiplets, closer to the expected multiplet ratio based on cell compositions and scRNA-seq studies [15]. Given the success of AMULET for identifying multiplets from snATAC-seq data with sufficient sequencing depth, this method can also be employed on the ATAC-seq component of multiomic assays to detect multiplets, where transcriptomes and epigenomes from the same nuclei are profiled simultaneously [18].

Epigenomic signal at marker peaks can be effectively used to annotate the cellular identities of the detected multiplets, as we achieved 85% accuracy on average in our simulations. Annotation of detected multiplets showed that the majority are from the same cell type. Furthermore, the likelihood of a multiplet to include nuclei from a certain cell type significantly correlated with the abundance of that cell type in the biospecimen. Since cells are lysed and nuclei are captured and profiled in snATAC-seq protocols [3], these assays likely do not contain many biological multiplets (i.e., due to cell-cell interactions) and multiplets were more likely to occur randomly among all nuclei. Hence, the most abundant cells are the most likely to form multiplets.

## Conclusions

Multiplets are inevitable in current droplet-based single-cell sequencing platforms, and their removal is essential for precise analyses and understanding of biological phenomena. AMULET is a novel and effective read count-based solution to detect and annotate multiplets from snATAC-seq data. Compared to simulation-based ArchR, AMULET had higher precision (0.61 vs. 0.28) assessed via donor demultiplexing. Furthermore, when snATAC-seq samples were sequenced to adequate depths (e.g., 25K valid reads per nucleus), AMULET achieved a very high recall (e.g., 0.85 in PBMCs) compared to ArchR (0.24 in PBMCs) while detecting simulated multiplets.

AMULET is a fast and efficient tool that can detect multiplets with a runtime that scales near linearly with the number of cells/valid reads (Additional file 1: Figure S11a,b), while requiring less than 3GB of memory (Additional file 1: Figure S11c,d). These analyses suggest that for most samples, AMULET will generate results within hours using a standard laptop. Code and documentation is freely available under a GPL V3 license at https://ucarlab.github.io/AMULET/, providing step by step guides and vignettes to easily integrate AMULET outputs into the frequently used Signac [11], Seurat [12, 13], and ArchR [7] pipelines.

## Methods

### PBMC1 and PBMC2 samples from human subjects

All studies were conducted following approval by the Institutional Review Board of UConn Health Center (IRB Number: UCONN IRB#16-071J-1). Following informed consent, blood samples were obtained from 2 healthy older volunteers (65 years and older) residing in the Greater Hartford, CT, USA region recruited by the UConn Center on Aging Recruitment and Community Outreach Research Core (http://health.uconn.edu/aging/research/research-cores/). Subjects were carefully screened to exclude potentially confounding diseases and medications, as well as frailty. Individuals who reported chronic or recent (i.e., within 2 weeks) infections were also excluded. Subjects were deemed ineligible if they reported a history of diseases such as congestive heart failure, ischemic heart disease, myocarditis, congenital abnormalities, Paget’s disease, kidney disease, diabetes requiring insulin, chronic obstructive lung disease, emphysema, and asthma. PBMCs were isolated from fresh whole blood using Ficoll-Paque Plus (GE) density gradient centrifugation.

### Human islet procurement

Human islets from two de-identified cadaveric organ donors were obtained through partnerships with the Integrated Islet Distribution Program (IIDP, http://iidp.coh.org/). Human islet functionality was assessed by static incubation glucose stimulated insulin secretion (GSIS) assays on the day after arrival, according to the IIDP protocol. Primary human islets were cultured in Prodo media (PIM-S + supplements PIM-G + PIM-ABS) in 5% CO_2_ at 37°C for approximately 24 h prior to dissociation and preparation for snATAC-seq profiling. To dissociate islets into single-cell suspension, 1ml of StemProAccutase (Thermo Fisher Scientific) per 1000 islet equivalent (IEQ, 1 IEQ=~1000 cells) was added, and cells were incubated for 10 min at 37°C with periodic pipetting at 2-min intervals. Islet single-cell suspension was washed three times in PBS + 0.03% BSA and passed through a 20-μm mesh filter to remove clumped cells and debris. Cell number was determined using Countess II FL Automated Cell Counter (Life Technologies).

### Islet and PBMC snATAC-seq cell labeling, capture, library preparation, and sequencing

For single nucleus ATAC sequencing (snATAC-seq) experiments, viable single-cell suspensions from each sample were used to generate snATAC-seq data using the 10X chromium platform according to the manufacturer’s protocols (Demonstrated Protocol Nuclei Isolation for ATAC Sequencing Document CG000169; Chromium Single Cell ATAC_User Guide RevB Document CG000168). Briefly, >100,000 cells from each sample were centrifuged and the supernatant was removed without disrupting the cell pellet. Lysis buffer was added for 5 min on ice to generate isolated and permeabilized nuclei, and the lysis reaction was quenched by dilution with Wash Buffer. After centrifugation to collect the washed nuclei, diluted nuclei buffer was used to re-suspend nuclei at the desired nuclei concentration as determined using a Countess II FL Automated Cell Counter and combined with ATAC buffer and ATAC enzyme to form a transposition mix. Transposed nuclei were immediately combined with Barcoding Reagent, Reducing Agent B and Barcoding Enzyme and loaded onto a 10X Chromium Chip E for droplet generation followed by library construction. The barcoded sequencing libraries were subjected to bead clean-up, checked for quality on an Agilent 4200 TapeStation, quantified by qPCR (KAPA Biosystems Library Quantification Kit for Illumina platforms), and pooled for sequencing on an Illumina NovaSeq 6000 S2 flow cell (2x50bp libraries).

### Pooled PBMC snATAC-seq data from three donors

Cryopreserved PBMCs isolated from three healthy unrelated donors were obtained from Hemacare (Los Angeles, CA, USA). PBMCs were thawed and cultured in RPMI 1640 with GlutaMax and HEPES (ThermoFisher) supplemented with 10% FBS and 1% pen/strep overnight on ultra-low-attachment 10cm dishes (Corning). Following overnight culture, suspension PBMCs were transferred to a 50mL conical tube and placed on ice. Adherent PBMCs were lifted using TrypLE and pooled with the suspension PBMCs. The cells were then washed twice with ice-cold PBS before nuclei isolation according to the “Nuclei Isolation for Single Cell ATAC Sequencing” protocol from 10x Genomics. Isolated nuclei were then resuspended in ice-cold 10x Genomics diluted nuclei buffer and counted. 15,000 nuclei from each PBMC donor were then subjected to transposition according to the “Chromium Next GEM Single Cell ATAC Reagent Kits v1.1” protocol from 10x Genomics. After transposition, nuclei from each donor were pooled, pelleted, resuspended in 10x Genomics ATAC Buffer B, and counted prior to microfluidic capture (targeting ~10,000 recovered nuclei). Next-generation sequencing library preparation was then performed according to supplier recommendations before sequencing on a single lane of an Illumina NovaSeq SP100 flow cell.

### snATAC-seq data processing, alignment, and nuclei clustering

snATAC-seq data was processed and aligned to the hg38 (PBMC1 and PBMC2) and hg19 (islet 1, islet 2, pooled PBMC) reference genomes using Cell Ranger ATAC pipeline (10x Genomics Cell Ranger ATAC 1.2.0). Position-sorted alignment files and barcodes passing QC (i.e., barcodes marked as cells) from Cell Ranger ATAC were then provided as input for AMULET. Nuclei were clustered using their accessibility profiles based on a two-pass clustering method previously described [3] (https://github.com/UcarLab/snATACClusteringPipeline), with two notable differences. First, we restrict the number of top bins (2.5kb in length) in the first pass clustering to the top 50k bins, up from 20k bins. Second, for second pass clustering, we increase the number of peaks to include all peaks identified in pass 1 up to 200k.

### Identifying snATAC-seq sites with >2 reads

Position sorted, paired-end read alignments from snATAC-seq data were compared to detect all sites with >2 unique reads per nucleus. To avoid instances where reads overlap due to technical reasons, we removed all read pairs that were marked using the following criteria available from the HTSJDK [19] library: (1) *ReadPairedFlag* = True, (2) *ReadUnmappedFlag* = False, (3) *MateUnmappedFlag* = False, (4) *SecondaryOrSupplementary* = False, (5) *DuplicateReadFlag* = False, and (6) *ReferenceIndex* != *MateReferenceIndex* (i.e., read pairs map to the same chromosome). Overlaps due to alignment errors were reduced by excluding reads based on (i) mapping quality scores less than or equal to 30 and (ii) insert sizes (i.e., the end-to-end distance between 5′ and 3′ read positions) greater than 900bp (~6 nucleosomes) in length.

To identify instances of >2 reads overlapping at any specific site, all intervals were identified for which an overlap was observed for at least two valid read pairs. Reads defining each interval were then compared to one another to identify all subintervals that exceed the specified overlap threshold (i.e., 2). To efficiently identify these subintervals, for each subset, interval breakpoints were defined at the start and end positions of each paired-end read. For each interval breakpoint, an integer value of 1 was assigned to all breakpoints originating from start positions, and −1 to all breakpoints originating from an end position. Interval breakpoints were then visited in position sorted order to generate a cumulative sum based on the assigned values at each breakpoint. The cumulative sum indicates the total number of overlaps between two interval breakpoints and efficiently identifies all sub-intervals with a number of overlaps greater than the specified threshold. Once all subintervals satisfying the threshold (i.e., 2) were identified for a subset of reads, the algorithm repeated this process for the remaining paired-end read subsets.

Identifying the initial intervals from coordinate sorted reads was performed using a linear time algorithm (i.e., O(n), *n* is the number of total reads), with an additional O(log(m)) (*m* equals the number of nuclei) overhead to identify their respective nucleus origin, resulting in O(n*log(m)) runtime. Identifying subintervals includes a sorting procedure that is dependent on the number of reads overlapping in the initial interval of overlapping reads identified. In the theoretical worst case, all reads overlap one another, resulting in O(n*log(n)) time to sort the start and end positions. In practice, the algorithm will run closer to n*log(m) time, as there are fewer instances of overlapping reads than total reads. AMULET assumes that reads are sorted beforehand and is otherwise superseded by the time necessary to sort reads by their chromosome and start positions (i.e., O(n*log(n)).

### Statistical detection of multiplets from snATAC overlap counts

Sites with >2 reads were first filtered using simple repeats, segmental duplications, repeat masker, and exclusion regions obtained from UCSC Genome Browser [20] and ENCODE [21, 22]. Next, filtered sites from all nuclei were merged if they overlapped by at least one base pair. Using this unified list of filtered sites with >2 reads, a binary matrix was generated where rows in the matrix represent sites with >2 reads for at least one nucleus, and the columns represent the individual nuclei within the sample. Values within the matrix were assigned to 1 if the nucleus and genomic site combination observed >2 reads overlapping, and 0 otherwise. From this matrix, multiplets can be detected using column sums (i.e., the total number of >2 read overlap instances for each nucleus) while repetitive element sequences can be inferred using row sums (i.e., the total number of cells observing >2 reads at the same genomic region).

Observing >2 reads overlapping across multiple sites within the same nucleus (or within the same site for multiple nuclei for inferring repetitive regions) can be modeled using the Poisson distribution. Occurrences of >2 reads overlapping are independent, counted within set intervals (i.e., counting sites across the entire genome within cells or counting nuclei within the same genomic site), are either present or not within these intervals, and have a constant average rate of occurring, satisfying the assumptions of the Poisson distribution. We therefore detected significant multiplets and inferred repetitive sequences with the Poisson cumulative distribution function, using respective mean row and column sum counts as the expected values to calculate Poisson probabilities. In this process, we first used Poisson probabilities to infer repetitive sequences (FDR < 0.01) where a significant number of nuclei observe >2 reads at the same genomic site. All inferred repetitive sequence genomic regions were removed from further analysis. Next, we calculated the Poisson probability of observing more sites with >2 reads than expected in a nucleus (i.e., multiplets) using column sums. Poisson probabilities for both detecting repetitive sequences and multiplet detection were corrected using the Benjamini-Hochberg procedure to adjust for multiple hypothesis testing. Repetitive sequences and multiplets were predicted by selecting sites or nuclei with adjusted Poisson probabilities less than 0.01.

### Simulating artificial multiplets to measure multiplet detection performances

To measure recall for detecting multiplets, artificial multiplets were simulated by combining accessibility profiles of nuclei within each sample population tested. For each sample, cells were randomly selected equal to 5% of the total cell population and additively paired together to introduce artificial multiplets equivalent to 2.5% of the total population. Introducing 2.5% artificial multiplets ensured that they were not the majority compared to real multiplets (5–11% of cells across all samples) present in the data. For heterotypic and homotypic multiplet comparisons, cell pairs were randomly reselected until they formed heterotypic or homotypic multiplets based on cell type annotations for 10 runs of simulations each. Simulated multiplets used for measuring the number of valid read pairs per nucleus did not have restrictions based on cell type and were selected at 5% of the total nuclei in the sample for 100 repetitions, selecting nuclei at random (i.e., Additional file 1: Figure S9). Artificial multiplets were introduced by generating modified barcode mappings (i.e., singlecell.csv output from CellRanger for AMULET) or barcodes in fragment files (i.e., Cell Ranger Fragments file for ArchR [7]), which assigned artificial multiplet reads to the same cell identifier (i.e., the first nucleus in the pair).

### Multiplet annotation pipeline

Detected multiplets are annotated using clusters identified for snATAC-seq samples, merging them with respect to specific cell types present in the cell population. In our study, PBMC clusters were merged to represent CD4^+^ T, CD8^+^ T, Natural Killer (NK), myeloid, and B cells and islet clusters were merged to represent alpha, beta, delta, and ductal cells. Marker peaks for all cell type clusters with at least 150 cells were identified with the FindMarkers function in Seurat [12], using the logistic regression setting. For the sake of unison, the top 100 marker peaks are then identified for each cell type cluster based on Bonferroni's adjusted *p* value of average log fold changes.

To account for data sparsity in snATAC-seq data, aggregate read profiles are calculated for each cell and marker peak. Aggregate read profiles are found by taking average read counts for each cell’s 15 nearest neighbors using the top 50 singular value decomposition (SVD) components. The cumulative distribution function in R (i.e., ecdf) is then used to find the abundance of reads for each cluster’s marker peaks. Distribution values represent the percent of each cell type’s accessibility profiles present within the cell and are referred to as cell type association scores. In order to distinguish multiplet types (i.e., heterotypic or homotypic) singlet profiles were calculated for each cell type in the sample. For each cell type’s singlet cells, cell type association scores at every marker peak were averaged to find the representative score profile for that cell type. Multiplets that have a profile close to their abundant cell type’s singlet profile were classified as homotypic. Euclidean distance was used to measure the similarity between the profiles of multiplets and singlets. Mixture models were then fitted to the distances with the Mclust R package [23] to group the closeness of the multiplets to their corresponding cell type’s singlet profile. Multiplets in the group with largest distance to the singlet profile are considered heterotypic. Multiplets are then annotated using the top 1 (for homotypic) or 2 (for heterotypic) cell type association scores.

## Supplementary Information

**Additional file 1.** Supplementary figures.

**Additional file 2.** Review history.

## Data Availability

The PBMC1 and PBMC2 datasets generated and/or analyzed during the current study are not publicly available due to being protected data but are available from the dbGAP repository (Accession number phs002361) [24] upon request, https://www.ncbi.nlm.nih.gov/projects/gap/cgi-bin/study.cgi?study_id=phs002361.v1.p1. The islet 1, islet 2, and pooled PBMC datasets are publicly available from the Gene Expression Omnibus (GEO) repository (GSE165212 )[25], https://www.ncbi.nlm.nih.gov/geo/query/acc.cgi?acc=GSE165212. Immune cell datasets from mice analyzed during the current study are available from the Gene Expression Omnibus (GEO) repository (GSE173415 [16], GSE149622 [17]), https://www.ncbi.nlm.nih.gov/geo/query/acc.cgi?acc=GSE173415 and https://www.ncbi.nlm.nih.gov/geo/query/acc.cgi?acc=GSE149622. Documentation and source code files for the AMULET framework are freely available under a GPL V3 license on GitHub at https://github.com/UcarLab/AMULET [26]. Code and scripts used in this study have been deposited on Zenodo, DOI 10.5281/zenodo.5189588 [27].
