# Supplementary material for: AMULET: a novel read count-based method for effective multiplet detection from single nucleus ATAC-seq data
Source: Genome Biol. 2021 Sep 1;22:252. doi: 10.1186/s13059-021-02469-x (PMC8408950; doi:10.1186/s13059-021-02469-x)
Supplement: Supplementary file 1 — Additional file 1. Supplementary figures. [file 13059_2021_2469_MOESM1_ESM.pdf]

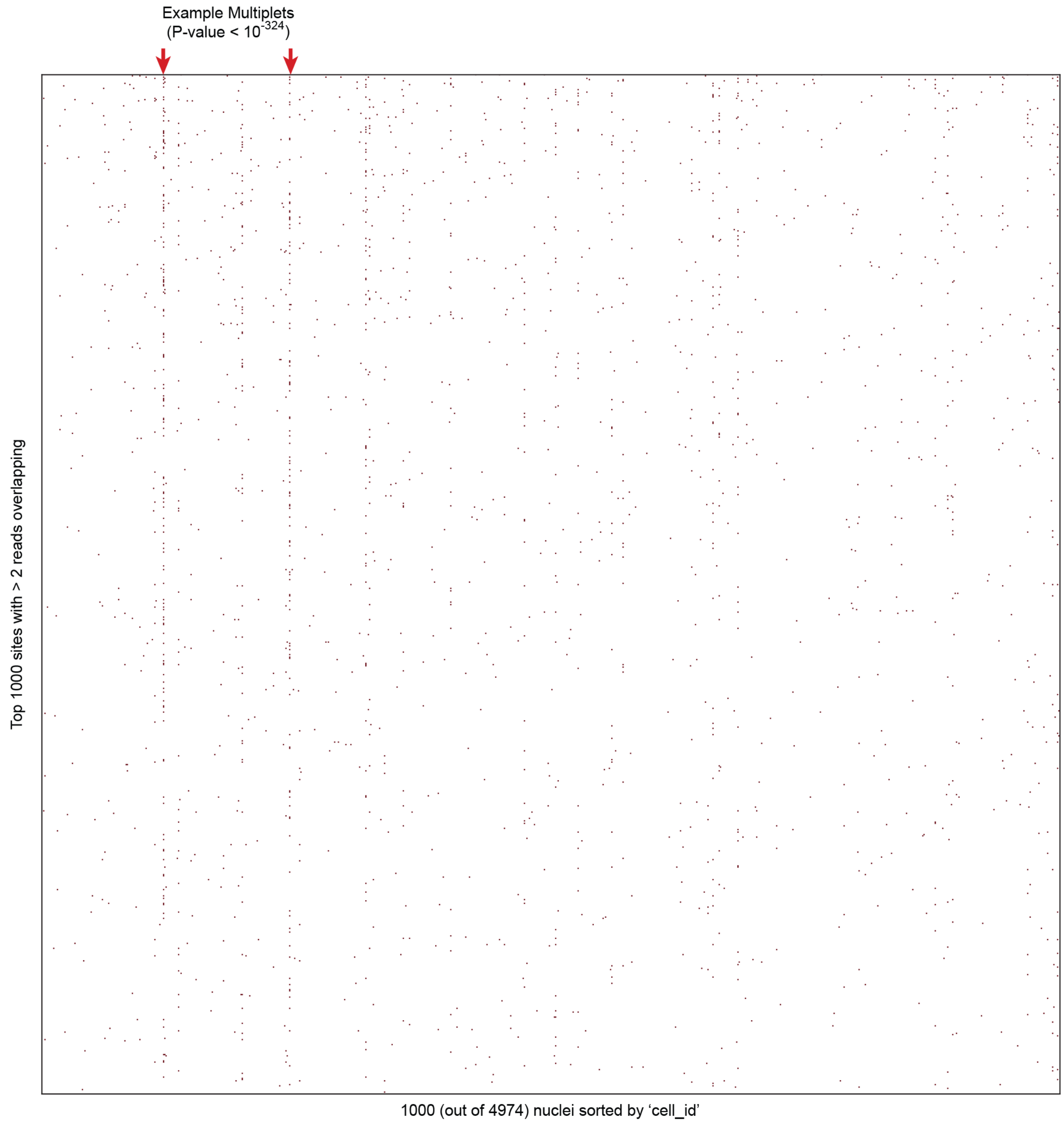

Figure S1: Multiplets observe many loci with >2 reads. The binary matrix of genomic regions with >2 reads per nucleus reveals high confidence multiplets (marked by arrows) that harbor many genomic regions with >2 reads. These multiplets can be clearly seen compared to the other nuclei in the subset.

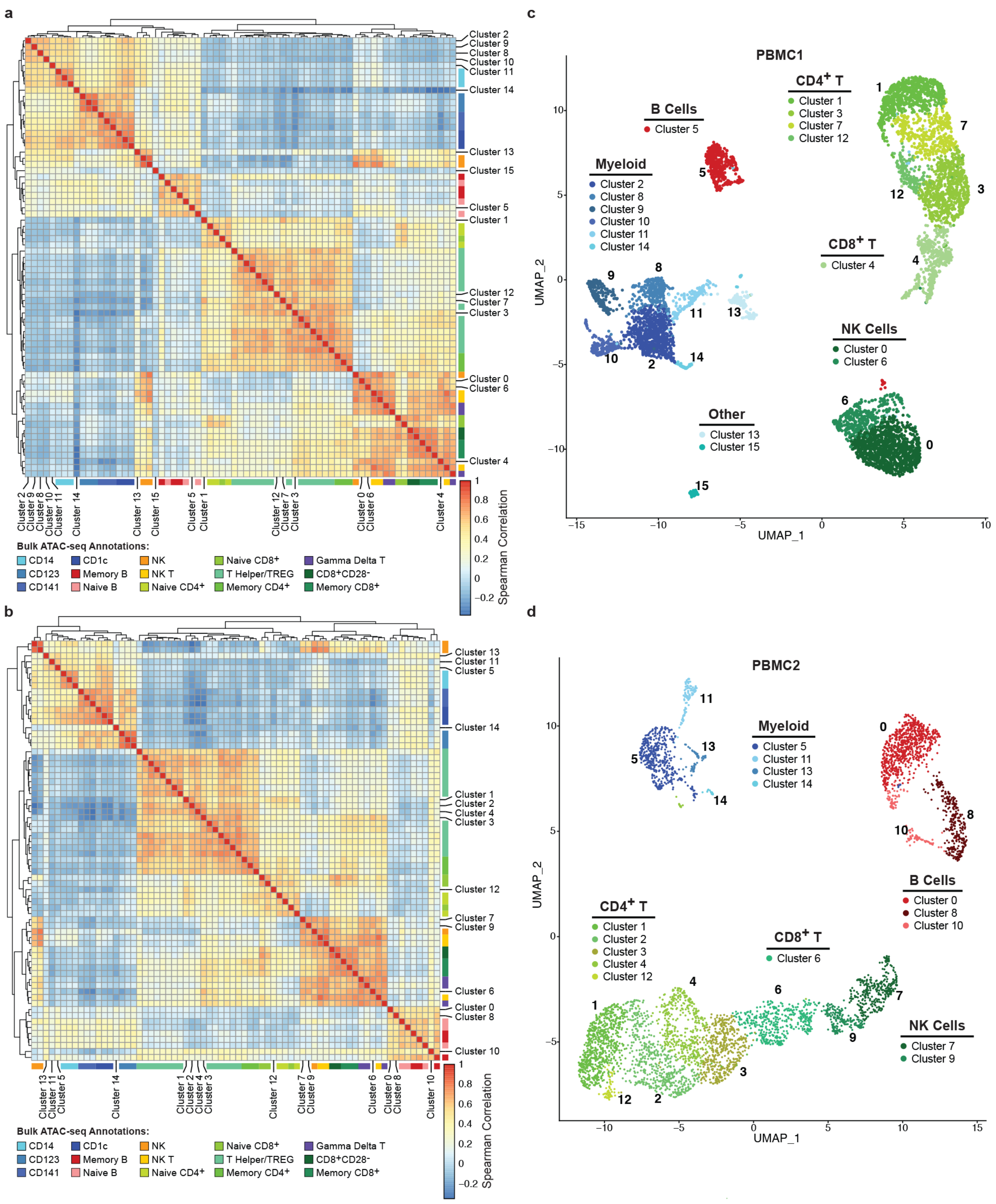

Figure S2: Pseudo-bulk snATAC-seq profile correlations with sorted bulk ATAC-seq revealed 5 major cell types. a, b, Spearman correlation heatmaps between pseudo-bulk (snATAC) and sorted bulk ATAC-seq accessibility profiles for PBMC1 (**a**) and PBMC2 (**b**). Pseudo-bulk profiles cluster with four major cell types: Myeloid, B, $CD4^{+}$ T, $CD8^{+}$ T and Natural Killer (NK). **c, d**, Annotated UMAP clusters for PBMC1 (**c**) and PBMC2 (**d**). Myeloid and B cells form distinct clusters for both samples. $CD4^{+}$T, $CD8^{+}$T and NK cell types share more accessible loci and tend to cluster more closely to one another.

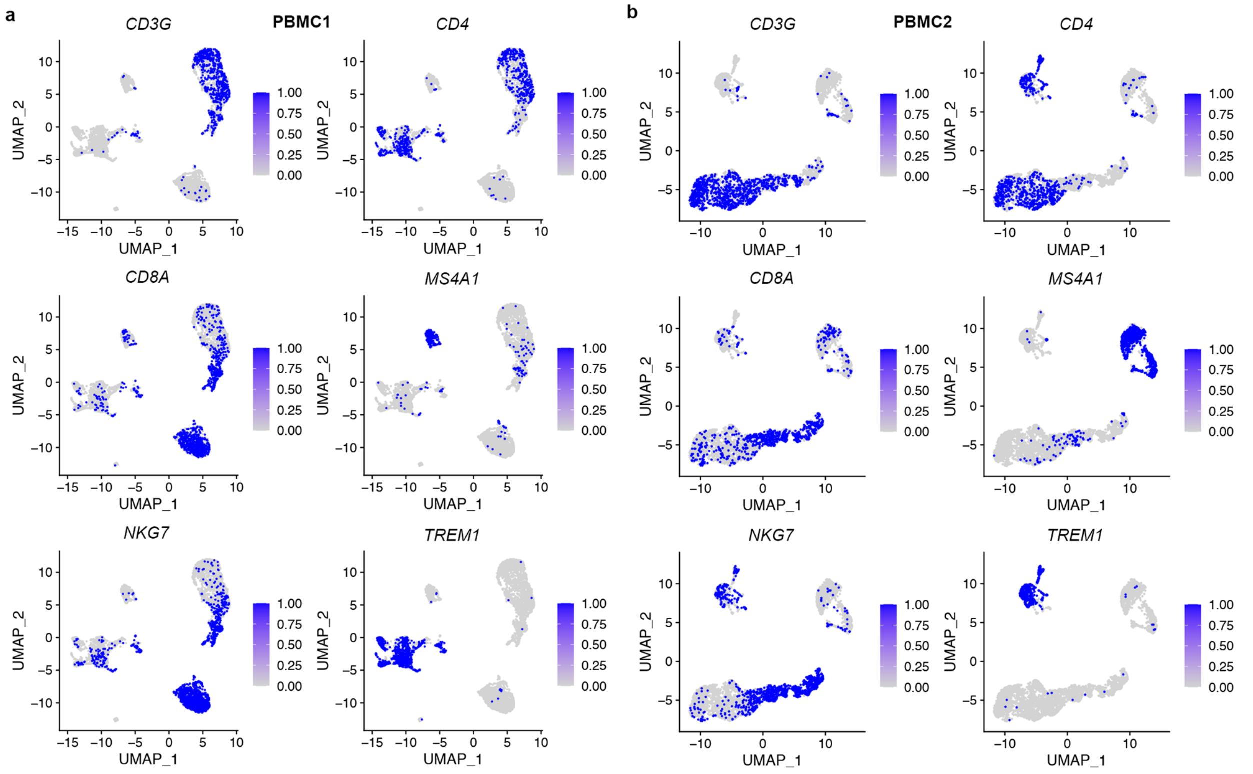

Figure S3: Annotated snATAC-seq clusters reflect accessibility at cell specific promoters. a, **b**, Annotated UMAPs for PBMC1 (**a**) and PBMC2 (**b**) at the promoters of *CD3G* (T-Cell Marker), *CD4* ($CD4^{+}$ T cell marker), *CD8A* ($CD8^{+}$ T cell marker), *MS4A1* (B cell marker), *NKG7* (NK cell marker), and *TREM1* (Myeloid cell marker). Accessibility was binarized to 0 or 1 based on the presence or absence of a read within these promoters. Using these markers, B and Myeloid cell types are clearly annotated with their respective markers. $CD4^{+}$ T and $CD8^{+}$ T cells can be observed by combining *CD3G* with *CD4* and *CD8A* markers respectively whereas NK cells can be seen using *NKG7* and excluding nuclei with accessibility at *CD3G* promoter.

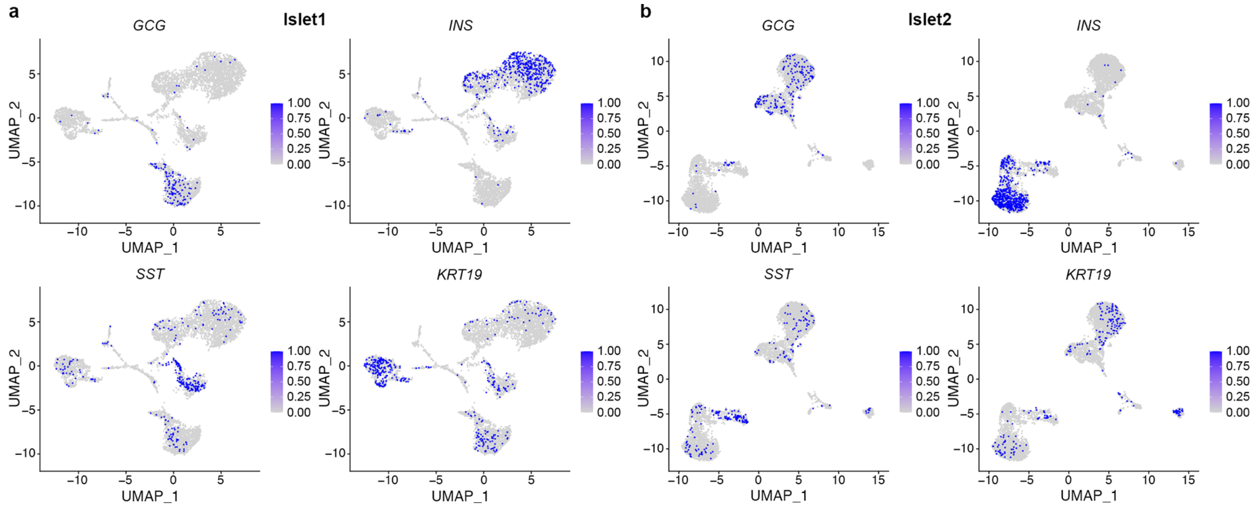

Figure S4: Islet snATAC-seq clusters correspond to cell marker annotations. a, **b**. Cell specific clusters correspond to their respective marker peaks for both islet 1(**a**) and islet 2 (**b**). Accessibility was binarized to 0 or 1 based on the presence or absence of a read within these promoters. Alpha, beta, delta and ductal cells are clearly identified with their respective marker genes: *GCG* (Alpha), *INS* (Beta), *SST* (Delta), and *KRT19* (Ductal).

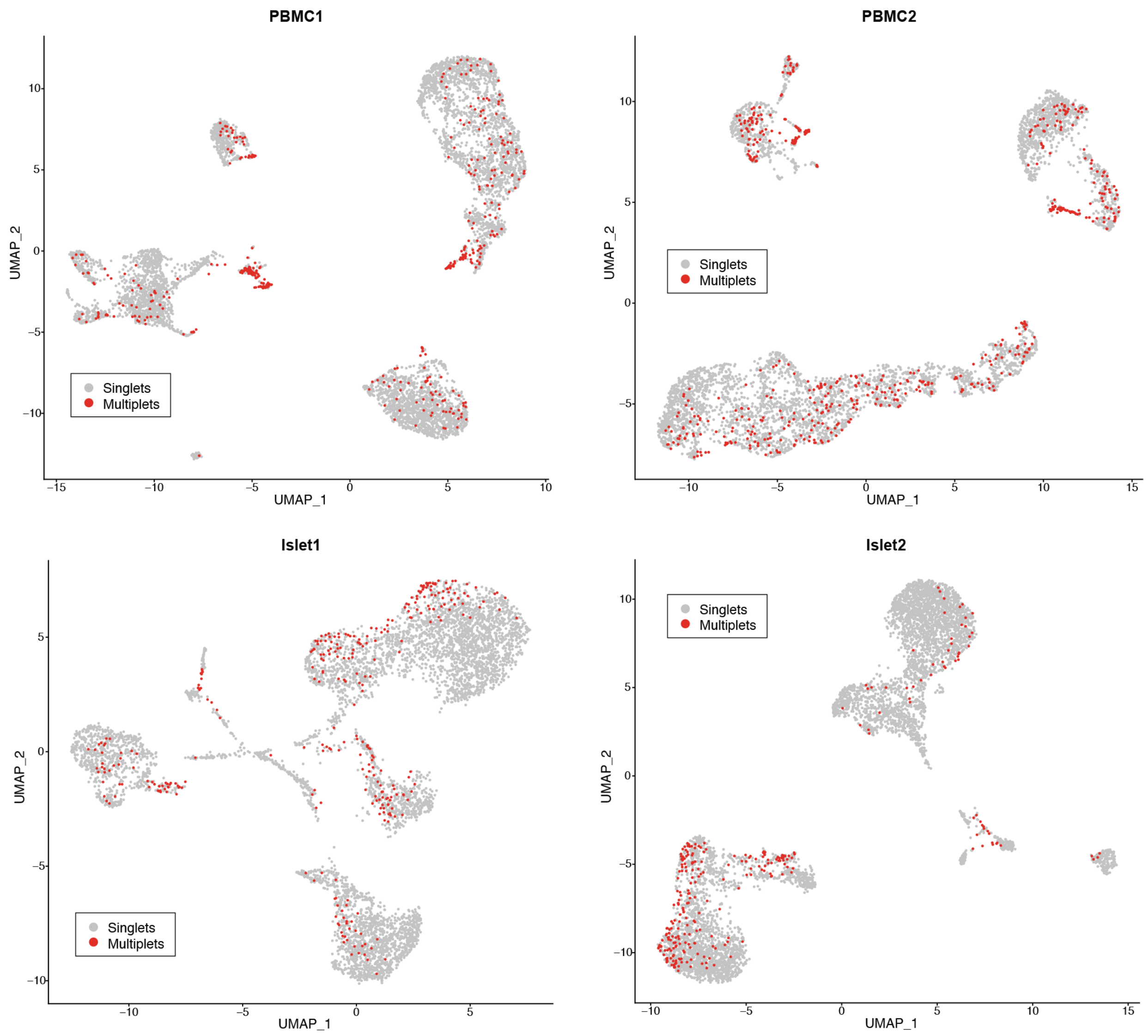

Figure S5: Multiplets are distributed throughout snATAC-seq clusters. Multiplet annotated UMAP clustering of PBMC1, PBMC2, islet 1 and islet 2 reveal that multiplets are distributed throughout all identified clusters and in some cases form their own multiplet clusters (i.e., center cluster in PBMC1). Multiplets between major cell type clusters are likely to be heterotypic.

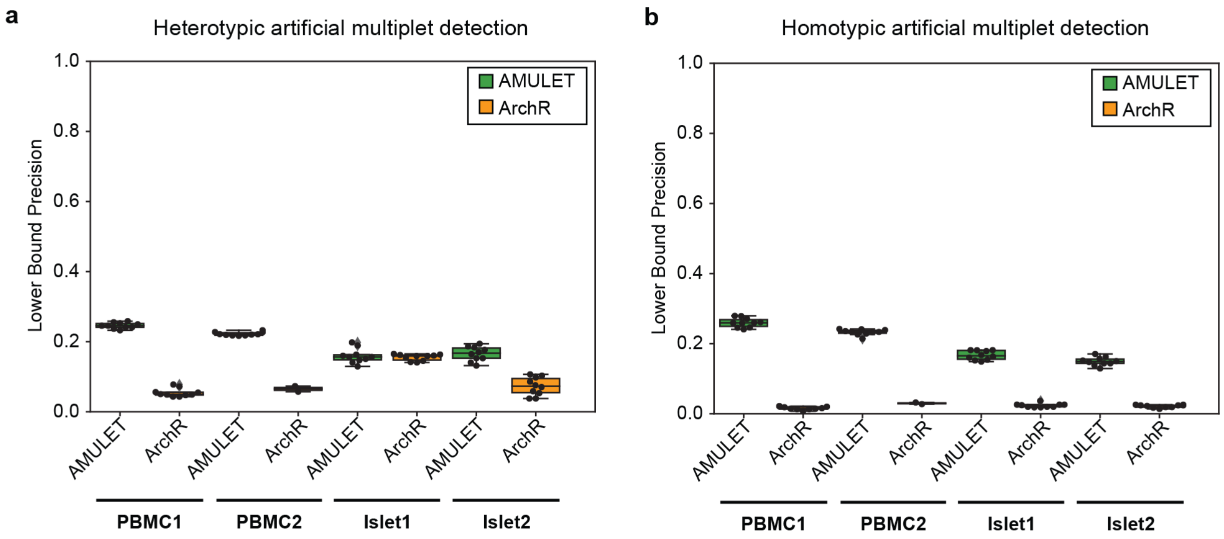

Figure S6: AMULET detects multiplets with higher lower bound precision than ArchR. Lower bound precision estimates for detecting simulated (**a**) heterotypic and (**b**) homotypic multiplets using AMULET and ArchR. In the worst-case scenario where multiplet calls other than simulated multiplets are false positives, AMULET observes higher precision compared to ArchR.

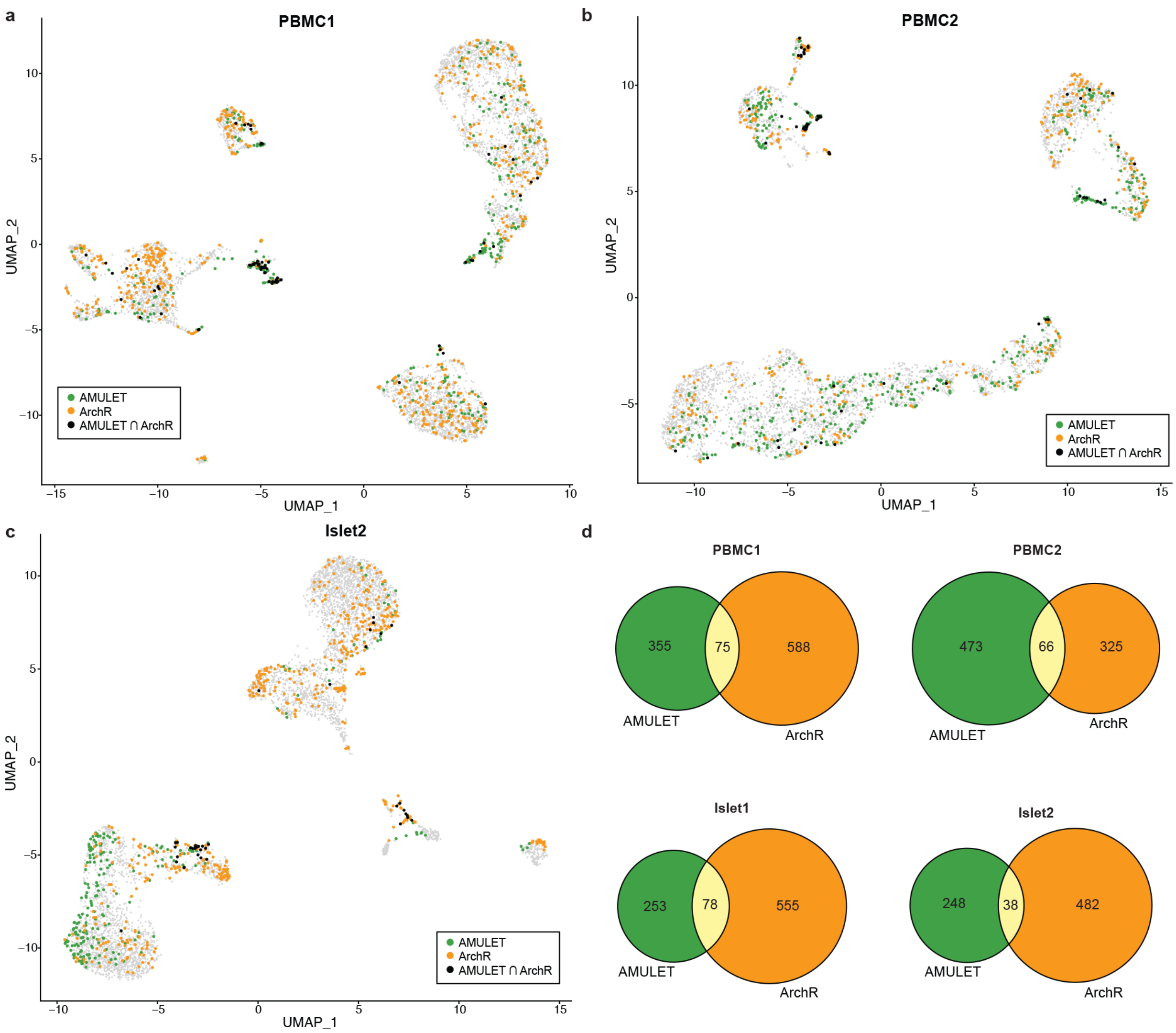

Figure S7: AMULET and ArchR identify different multiplet subsets. a-c, UMAP clusters annotating AMULET multiplets (green), ArchR multiplets (orange), or their intersection (black) for PBMC1 (**a**), PBMC2 (**b**), and islet 2 (**c**). Majority of multiplets detected by both AMULET and ArchR were between major cell type clusters (i.e., heterotypic multiplets). **d**, Comparison of multiplets detected by AMULET and ArchR. Only small subsets of multiplets are detected by both methods for each sample.

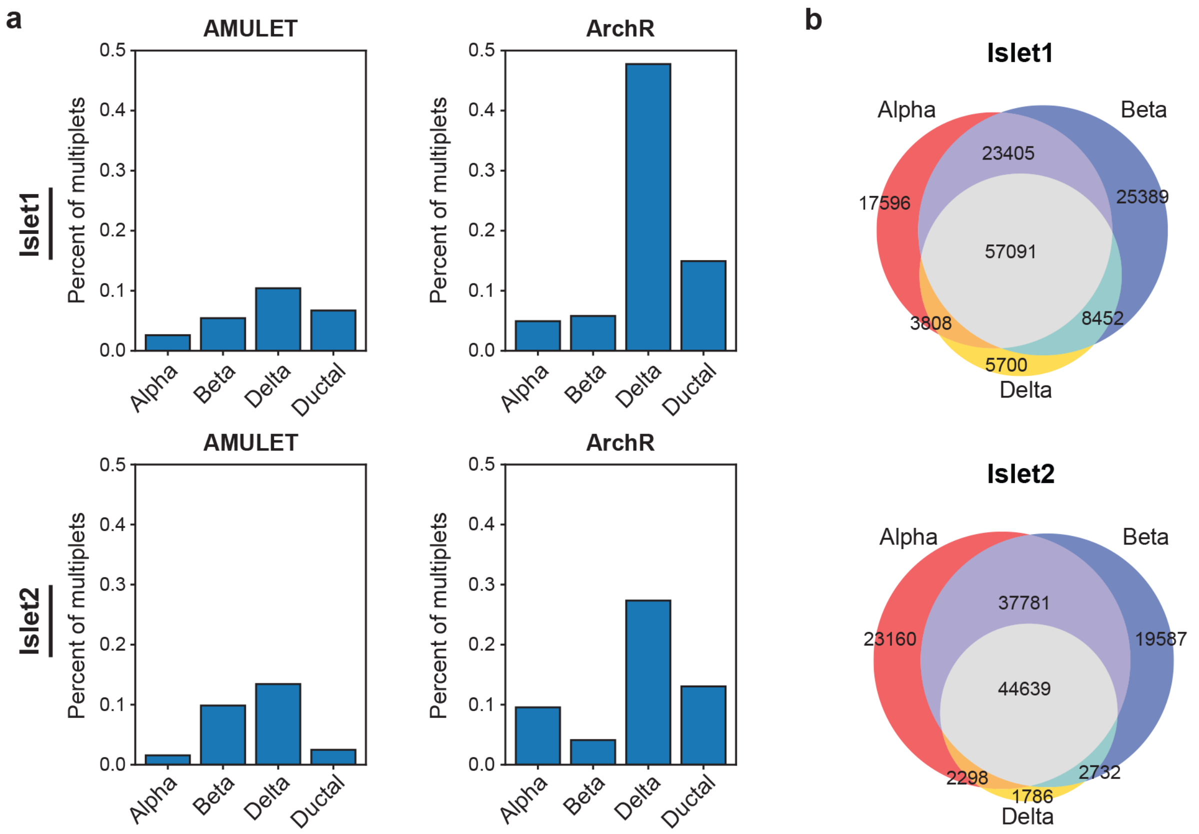

Figure S8: AMULET and ArchR multiplets comparisons reveal nature of their underlying algorithms. a, Percent of multiplets detected for each annotated cell type in islet 1 (top barplots) and islet 2 (bottom barplots). Between 27% (islet 2) and 47% (islet 1) of muliplets are Delta cells. **b**, Overlap of peaks called for respective Alpha, Beta, and Delta cell clusters using the unified list of peak calls used for cell clustering. Delta cells have fewer cell specific peaks than peaks shared with beta cells.

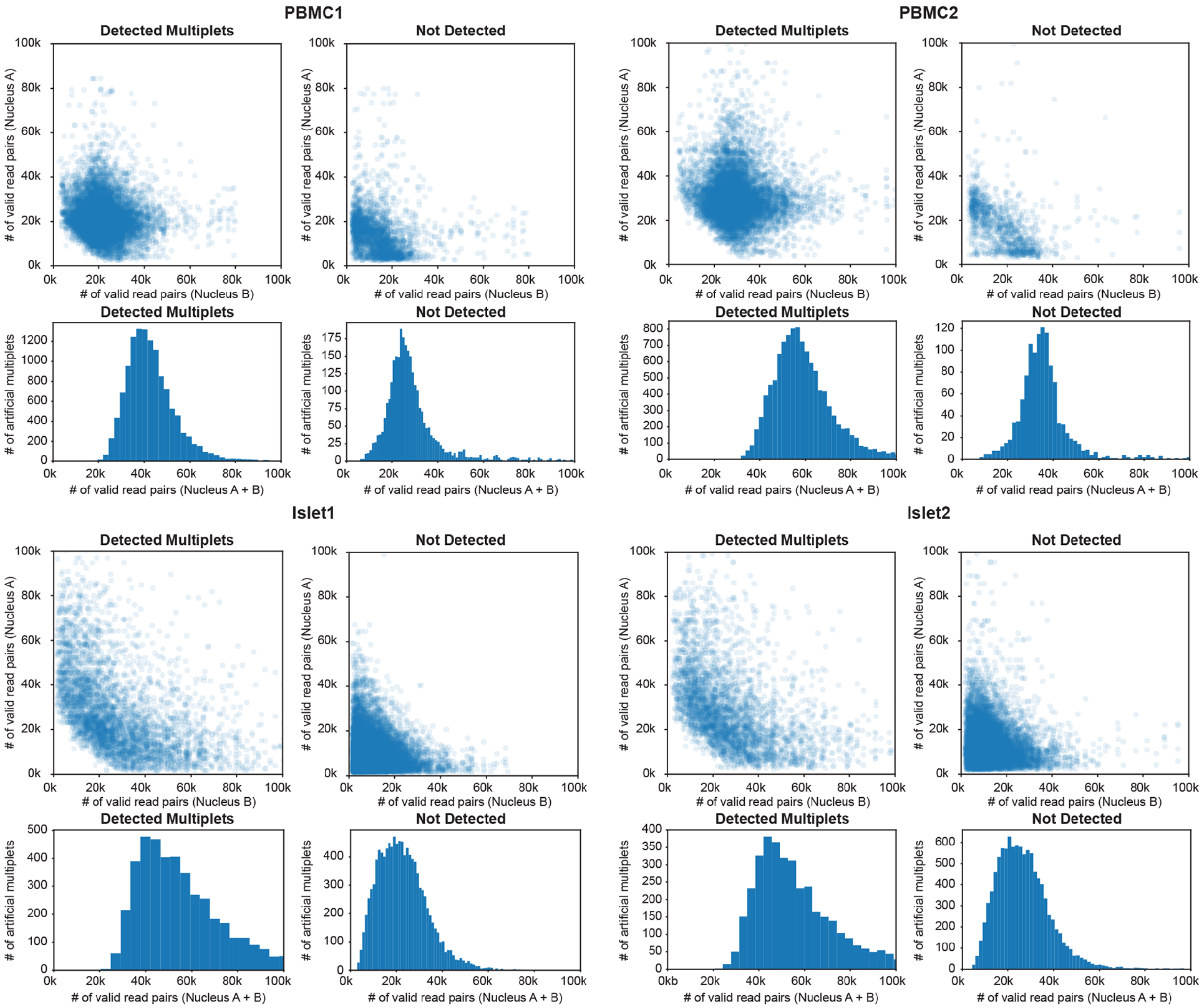

Figure S9: Artificial multiplets are detected when combined valid read pairs exceed 40K. For each sample, multiplets were detected (Top left for each sample) or not detected (Top right for each sample), depending on whether one or both nuclei exceeded 20K valid read pairs. Histogram of combined profiles revealed that the majority of detected multiplets (bottom left for each sample) had at least 20K valid read pairs while multiplets not detected were those with less than 40K valid read pairs (bottom right for each sample). When nuclei are sequenced for 20k valid reads per nuclei, multiplets will harbor 40K valid read pairs and can be detected by AMULET.

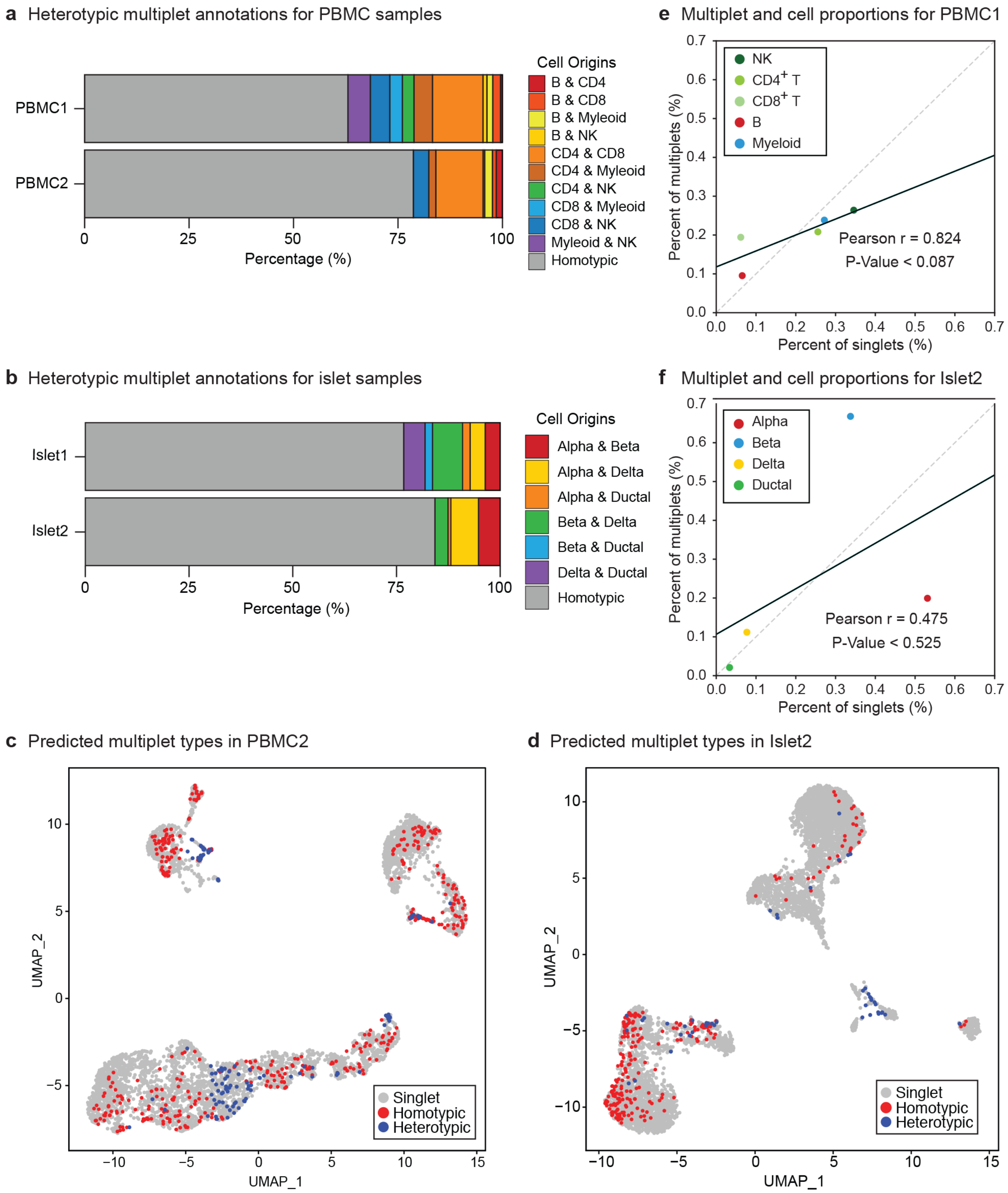

Figure S10: Multiplet annotations correspond to cell proportions. a,**b**, Heterotypic cell type annotations for PBMC (**a**) and islet (**b**) samples. Majority of multiplets are annotated as homotypic. **c-d**, UMAP clustering for heterotypic and homotypic multiplet annotations in PBMC2 (**a**) and islet 2 (**b**). Heterotypic multiplets are found between major cell type clusters. Homotypic multiplets are observed on the periphery of major cell type clusters. **e**-**f**, Cell and multiplet proportions for PBMC1(**e**) and islet 2(**f**). Multiplet cell type proportions are highly correlated with overall cell proportions. islet 2 observed more beta cell multiplets than other cell types/samples, reducing correlation and significance for islet 2.

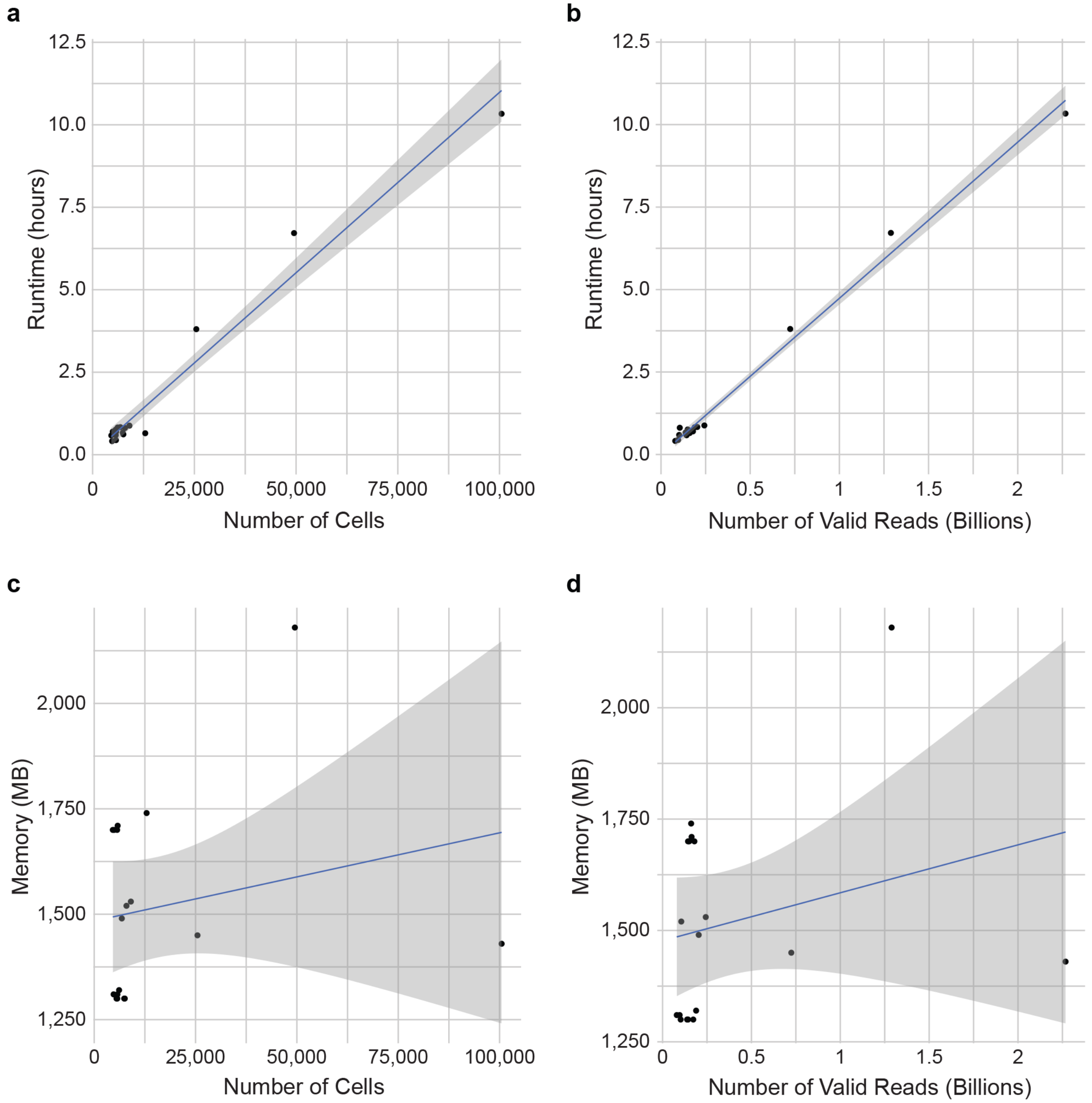

Figure S11: Runtime and memory requirements for running AMULET for different cell numbers and read counts. a,b, Runtime scales linearly with respect to the number of (**a**) cells and (**b**) valid read pairs, in concordance with the theoretical expectations based on the worst case runtime to sort the reads, O(n*nlog(n)). **c,d,** AMULET requires less than 3GB of memory for up to 100k cells (**c**) and 2.5 billion valid read pairs (**d**).
