# Supplementary material for: AMULET: a novel read count-based method for effective multiplet detection from single nucleus ATAC-seq data
Source: Genome Biol. 2021 Sep 1;22:252. doi: 10.1186/s13059-021-02469-x (PMC8408950; doi:10.1186/s13059-021-02469-x)
Supplement: Supplementary file 2 — Additional file 2. Review history. [file 13059_2021_2469_MOESM2_ESM.docx]

Review History

**First round of review**

**Reviewer 1**

**Were you able to assess all statistics in the manuscript, including the appropriateness of statistical tests used?**

Yes: I am able to assess all statistics in the manuscript, including the appropriateness of the statistical tests used.

**Were you able to directly test the methods?**

No

**Comments to author:**

Thibodeau and Eroglu et al. describes a new strategy to detect doublet cells in single nucleus ATAC-seq datasets, alternative to the commonly used simulation-based methods. Their algorithm is based on the assumption that no more than two overlapping fragments at one locus should be observed in single cells. The authors then used Poisson distribution to model the number of regions with >2 uniquely aligned fragments in each cell/nucleus, and denote cells/nucleus with adjusted Poisson probability <0.01 as multiplets. The authors compared the performance of their method to ArchR (which is simulation-based) on two newly generated datasets (PBMC and pancreatic islets) with simulated doublets, and showed their recall is much better than ArchR. Overall, the doublet detection method proposed here is new and theoretically sound, however, there are critical issues with the paper which needs to be addressed or clarified before the reviewer is convinced that this method works.

1)	In the comparison with ArchR, the authors only showed the recall (percentage of the simulated doublets being captured) but did not report precision. High recall rate will not be useful if the precision is low. In this case, it is likely that this method captures many of the true doublets, but at the same time mis-labels a lot of singlets as doublets. The "precision" metric should be included in all comparisons for the reviewers to assess the performance of the method.

2)	As I said earlier, this algorithm is theoretically sound. However, in practice, it is compromised by the alignment errors in the genome (repetitive elements, imperfect genome assembly, etc) that result in >2 fragments in one place. The authors have acknowledged these potential sources of errors (Page3, Line 83), and tried to use annotations and inference from actual data (page 16, Line 359-367) to filter out these problematic regions. I can imagine this is one of the major hurdles for the proposed method to work well. However, it is unclear whether the filtering strategies the authors adopted have ameliorated or overcome such problem. It would be nice if the authors can use different cut-offs to flag the "problematic regions", and prove that their strategy indeed improve their performance of detecting the doublets. And again, both precision and recall should be included.

3)	I wanted to note that the method in theory will only work with diploid cells. For instance, it cannot be applied to snATAC-seq data generated from non-diploid genome, such as cancer samples or gametes, where the simulation-based methods can still be applied. Can the authors think of alternative strategies in these scenarios? At least I feel these limitations should be discussed in the manuscript.

4)	I'd like to commend the authors for creating a very detailed documentation for the software. I haven't been able to download and run the software myself (lack of test data in hand). I would recommend the users include a test dataset if possible.

**Reviewer 2**

**Were you able to assess all statistics in the manuscript, including the appropriateness of statistical tests used?**

Yes: I did not find any obvious issue.

**Were you able to directly test the methods?**

No

**Comments to author:**

In this manuscript the authors reported a new method for detecting "doublets" from single-cell ATAC-seq data sets. Single-cell or single-nucleus ATAC-seq is becoming a widely used method for profiling chromatin accessibility at a single-cell resolution, and for characterizing epigenetic heterogeneity in various tissue samples. Almost all such data sets contain various level of doublets that can complicate downstream analyses and sometime lead to wrong interpretation. While methods like ArchR has been developed for doublet detection, this is far from being a solved problem, since the detection accuracy has been lower than adequate. ATAC-DoubletDetector addresses this issue from an angle that hasn't been explored before, by using information of overlapping reads and formulating a statistical test for doublet detection. The algorithm was implemented in Java and made freely available on Github. The authors generated two sets of 10X scATAC-seq data from PBMC and pancreatic islets, and demonstrated a superior performance over ArchR, a widely used method which has a functionality for doublet detection. The recall rate on the denser data set (~20k unique reads per cell) is quite impressive. Even on the less dense data set, ATAC-DoubletDetector still compares favorably with ArchR. The author further implemented a functionality for annotating the doublets, and showed that most doublets, at least in their own data sets, are mostly formed randomly due to technical reasons, which is more or less expected. Overall I find this method useful in the field of single-cell analysis.

This manuscript can be further improved in the following areas.

(1) Clearly the performance of ATAC-DoubletDetector depends on the density of the data. Data sets that have higher unique reads per cells are more suitable for this method. The number of unique reads per cells depends on multiple factors, including the quality of samples, experimental procedures, and the cell or tissue types (some cell types have less open chromatin overall than others). The authors have reported some results on the two data sets that they generated. To provide the readers a better picture of a sweet spot (or zone) for the method, two data points are a bit thin. The authors can perform down-sampling of the PBMC data set at different read density, and report the recall rates, perhaps in a plot. Further, the authors can check a number of recently published 10X scATAC-seq data from other groups, and place them on that plot, which will better inform what studies might be benefited from ATAC-DoubletDetector. A plot like that can clear reveal how broad the impacts this method will make.

(2) For the potential users, it would be helpful to report the computational needs, for example, CPU hours, RAM, run time on typical data set of different cell numbers, such as 5,000, 10,000, or even 100k.

**Authors Response**

**Point-by-point responses to the reviewers’ comments:**

*We thank the reviewers for their suggestions and feedback. We have included additional data and analyses to address their comments, which significantly improved the manuscript and the associated tool. We also renamed our method AMULET (ATAC-seq MULtiplet Estimation Tool), which we believe is a more appropriate name. Below we summarize our point-by-point responses to reviewers’ comments.*

Reviewer 1 Comments:

Reviewer 1: “Thibodeau and Eroglu et al. describes a new strategy to detect doublet cells in single nucleus ATAC-seq datasets, alternative to the commonly used simulation-based methods. Their algorithm is based on the assumption that no more than two overlapping fragments at one locus should be observed in single cells. The authors then used Poisson distribution to model the number of regions with >2 uniquely aligned fragments in each cell/nucleus, and denote cells/nucleus with adjusted Poisson probability <0.01 as multiplets. The authors compared the performance of their method to ArchR (which is simulation-based) on two newly generated datasets (PBMC and pancreatic islets) with simulated doublets, and showed their recall is much better than ArchR. Overall, the doublet detection method proposed here is new and theoretically sound, however, there are critical issues with the paper which needs to be addressed or clarified before the reviewer is convinced that this method works.”

*We thank reviewer 1 for finding our method novel and theoretically sound.*

Reviewer 1: “In the comparison with ArchR, the authors only showed the recall (percentage of the simulated doublets being captured) but did not report precision. High recall rate will not be useful if the precision is low. In this case, it is likely that this method captures many of the true doublets, but at the same time mis-labels a lot of singlets as doublets. The "precision" metric should be included in all comparisons for the reviewers to assess the performance of the method.”

*Response: We agree with reviewer 1 that precision is an important metric to report. However, calculating the precision of multiplet detection methods is challenging due to not knowing which nuclei are true singlets (“true negatives”, TN) and which are true multiplets (“true positives”, TP). We have estimated the recall (TP/(TP+FN)) by treating the simulated multiplets as true positive examples. Unlike multiplets, where simulated multiplets are undoubtedly multiplets, estimating singlets is more challenging. Therefore, to estimate the precision (TP/(TP+FP)), we followed two strategies:*

*1) We estimated the lower bound for precision by assuming the worst-case scenario (i.e., all nuclei that are detected by the algorithm and not simulated as a multiplet is a false positive (FP) call). Accordingly, AMULET had a higher average lower bound precision than ArchR for both heterotypic (0.20 versus 0.09) and homotypic (0.20 versus 0.02) multiplets (Response Figure 1). Note that in the best-case scenario (i.e., all detected multiplets are indeed multiplets), both methods have perfect precision =1. Obviously, the exact precision lies between the worst- and best-case scenarios; however, it is not possible to estimate it exactly without further information. As suggested by the reviewer, we have included these lower bound precision calculations as supplementary information (Additional File 1: revised Figure S6).*

*2) As an empiric attempt to more precisely address this question, we included new snATAC-seq data from collaborators (Dr. Gartner and his team members, who are now co-authors) from the PBMCs of 3 individuals that were mixed and sequenced together. These samples were demultiplexed based on the individuals’ genetic variation using Vireo[1]. This pooled design facilitates the identification of multiplets that comprised of cells from different individuals (i.e., cells that harbor genetic variants from two or three individuals). Although this design will not uncover all possible multiplets (e.g. within-donor multiplets may be missed), it will identify true multiplets among donors with high confidence that can be used to estimate the precision of AMULET and ArchR predictions. Using Vireo, we detected 244 true multiplets among donors out of 3,812 cells (Response Figure 2a). AMULET detected 67-84 multiplets from these data at different FDR cutoffs (1,5,10%), whereas ArchR detected 175 multiplets using their default/recommended parameters (Response Figure 2b-d). Based on the multiplets identified by Vireo, we calculated the precision and recall of each method. Accordingly, AMULET had much higher precision compared to ArchR (0.57-0.61 versus 0.28) while the recall values were similar (0.17-0.20 for AMULET versus 0.20 for ArchR, Response Figure 2d). The low recall likely stems from the low depth of sequencing for these samples (average read count= 7,683 valid read pairs per nucleus). These new results are included in the manuscript as revised Figure 3.*

*Together these new analyses and data demonstrate that read-count based AMULET exhibits high precision (lower false positives) even when the recall is low due to read depth, significantly outperforming the simulation-based alternative ArchR. We have included these new data and analyses in the manuscript as an additional section (lines 149-173):*

*“High recall may result from many false positive multiplet calls. Therefore, it is also critical to assess precision, which quantifies the number of positive class predictions that actually belong to the positive class (i.e., the ratio of true multiplets detected by the method). However, calculating precision is challenging without ground-truth singlet and multiplet classifications. Here, we estimated the lower bound for precision by assuming the worst-case scenario; i.e., all nuclei that are called as multiplets and not simulated as one are false positive calls. Accordingly, even in the worst-case scenario AMULET had a higher average precision than ArchR for heterotypic (0.23 versus 0.06 for PBMCs, 0.16 versus 0.11 for Islets) and homotypic (0.25 versus 0.02 for PBMCs, 0.16 versus 0.02 for Islets) cases (Additional file 1: Figure S6). Note that the best-case scenario for precision assumes that all detected multiplets are true positives; hence the upper bound for precision for both methods and for all datasets will be 1 (i.e., perfect precision).*

*To better estimate the precision of multiplet calls, we generated multiplexed snATAC-seq data from the PBMCs of 3 individuals that were pooled and sequenced together. These samples were demultiplexed using Vireo[10] based on naturally occurring genetic variation in each individual, which also effectively detects multiplets among donors. Although multiplets among donors are a subset of all true multiplets, these data still enable an unbiased framework to benchmark different methods in terms of their precision. By using Vireo, we detected 244 true multiplets formed from the nuclei of different donors out of 3,812 nuclei (Fig. 3c), providing an unbiased ground truth to estimate precision for donor multiplets. We then applied AMULET (FDR 1%, 5%, 10%) and ArchR (default parameters) to these data to detect 67-84 and 175 multiplets, respectively. 59% of AMULET multiplets (at 5% FDR) were also Vireo multiplets (Fig. 3d), while only 28% of ArchR multiplets were also identified by Vireo (Fig. 3e). Accordingly, AMULET detected Vireo multiplets with higher precision (0.57-0.61) compared to ArchR (0.28); both methods achieved a similar recall, 0.17-0.20 and 0.20 respectively (Fig. 3f).*

*Together, these results suggest that read-count based AMULET can detect multiplets with high precision (lower false positives assessed by sample multiplexing) and high recall (lower false negatives assessed by simulated multiplets), especially when samples are sequenced deeply (e.g., 20K-28K average, 19K-28K median valid read pairs for PBMC1 and PBMC2), serving as an effective alternative to simulation-based methods.“*

Reviewer 1: As I said earlier, this algorithm is theoretically sound. However, in practice, it is compromised by the alignment errors in the genome (repetitive elements, imperfect genome assembly, etc) that result in >2 fragments in one place. The authors have acknowledged these potential sources of errors (Page3, Line 83), and tried to use annotations and inference from actual data (page 16, Line 359-367) to filter out these problematic regions. I can imagine this is one of the major hurdles for the proposed method to work well. However, it is unclear whether the filtering strategies the authors adopted have ameliorated or overcome such problem. It would be nice if the authors can use different cut-offs to flag the "problematic regions", and prove that their strategy indeed improve their performance of detecting the doublets. And again, both precision and recall should be included.

*To address the reviewer’s comment, we have applied AMULET on 8 different samples in humans and mice[2,3] with different parameter settings and cutoffs for minimum mapping quality (>0, >10, >20, >30), inferred repetitive element filtering q-value (<0.01, <0.05, <0.1), and user provided problematic regions (with or without) (Response Figure 3). First, we observed that the number of multiplets detected in each sample remained relatively the same for different parameter settings suggesting that AMULET multiplet predictions are robust to the inclusion/exclusion of these problematic regions (Response Figure 3a). We also calculated recall and lower bound precision at different settings for each sample using artificial multiplets and observed that the performance of the algorithm is consistent across different cutoffs and parameter settings in which the maximum difference in recall was 4% and the maximum difference in lower bound precision was 3% (Response Figure 3b,c). These results suggest that identifying multiplets based on the Poisson cumulative distribution function is robust to noise since problematic regions (even when they are not filtered out) are likely to be randomly accessible across all nuclei. Hence, they will not be significantly enriched in certain nuclei and will not bias multiplet calling. Although these analyses suggest that problematic regions have a minimal effect on multiplet calls, we still recommend these regions to be eliminated prior to multiplet calling.*

Reviewer 1: I wanted to note that the method in theory will only work with diploid cells. For instance, it cannot be applied to snATAC-seq data generated from non-diploid genome, such as cancer samples or gametes, where the simulation-based methods can still be applied. Can the authors think of alternative strategies in these scenarios? At least I feel these limitations should be discussed in the manuscript.

*Reviewer 1 is right; our method is developed for diploid cells. However, as the reviewer suggested, it would be possible to improve it to study non-diploid cells in the future. First, multiplets in haploid and polyploid cells can potentially be identified by adjusting the read count overlap threshold from 2 to the number of chromosomes in the studied cell type. A potential caveat of this approach for polypoid cells is that the samples need to be sequenced deeper to capture potential multiplets. In addition, in the future, AMULET can be improved to analyze snATAC-seq data from certain portions of the genome (i.e., tandem duplication of chromosomal segments in cancer). We have plans to improve the software in the future for such applications; we briefly discuss this caveat and the future directions in the revised manuscript (lines 258-261), also copied below.*

*“AMULET was designed to detect multiplets in diploid cells, by systematically searching for sites with >2 read counts. This method can be improved in the future to analyze snATAC-seq data from haploid cells (e.g., gametes), polyploid cells, or aneuploid cancer cells by adjusting the expected number of reads per genomic region parameter or restricting the analyses to specific portions of the genome.“*

Reviewer 1: I'd like to commend the authors for creating a very detailed documentation for the software. I haven't been able to download and run the software myself (lack of test data in hand). I would recommend the users include a test dataset if possible.

*We thank Reviewer 1 for appreciating our detailed documentation. We extended our method to also use the fragment files from CellRanger ATAC as input to detect multiplets as requested by some users. With this extended functionality, publicly available fragment files can be used as a test dataset. For example:*

*https://cf.10xgenomics.com/samples/cell-atac/1.0.1/atac_v1_pbmc_10k/atac_v1_pbmc_10k_fragments.tsv.gz*

*In addition, we also provide below advanced access to our GEO repository that includes snATAC-seq from human islets as well as multiplexed samples from human PBMCs. These changes provide early access to test data.*

*URL: https://www.ncbi.nlm.nih.gov/geo/query/acc.cgi?acc=GSE165212*

*Code: ohgjgwgevxgjfap*

Reviewer 2 Comments:

Reviewer 2: In this manuscript the authors reported a new method for detecting "doublets" from single-cell ATAC-seq data sets. Single-cell or single-nucleus ATAC-seq is becoming a widely used method for profiling chromatin accessibility at a single-cell resolution, and for characterizing epigenetic heterogeneity in various tissue samples. Almost all such data sets contain various level of doublets that can complicate downstream analyses and sometime lead to wrong interpretation. While methods like ArchR has been developed for doublet detection, this is far from being a solved problem, since the detection accuracy has been lower than adequate. ATAC-DoubletDetector addresses this issue from an angle that hasn't been explored before, by using information of overlapping reads and formulating a statistical test for doublet detection. The algorithm was implemented in Java and made freely available on Github. The authors generated two sets of 10X scATAC-seq data from PBMC and pancreatic islets, and demonstrated a superior performance over ArchR, a widely used method which has a functionality for doublet detection. The recall rate on the denser data set (~20k unique reads per cell) is quite impressive. Even on the less dense data set, ATAC-DoubletDetector still compares favorably with ArchR. The author further implemented a functionality for annotating the doublets, and showed that most doublets, at least in their own data sets, are mostly formed randomly due to technical reasons, which is more or less expected. Overall I find this method useful in the field of single-cell analysis.

*We thank Reviewer 2 for their positive comments and finding our method useful for single cell analyses.*

Reviewer 2: Clearly the performance of ATAC-DoubletDetector depends on the density of the data. Data sets that have higher unique reads per cells are more suitable for this method. The number of unique reads per cells depends on multiple factors, including the quality of samples, experimental procedures, and the cell or tissue types (some cell types have less open chromatin overall than others). The authors have reported some results on the two data sets that they generated. To provide the readers a better picture of a sweet spot (or zone) for the method, two data points are a bit thin. The authors can perform down-sampling of the PBMC data set at different read density, and report the recall rates, perhaps in a plot. Further, the authors can check a number of recently published 10X scATAC-seq data from other groups, and place them on that plot, which will better inform what studies might be benefited from ATAC-DoubletDetector. A plot like that can clear reveal how broad the impacts this method will make.

*We thank Reviewer 2 for this suggestion. We did run a thorough analyses in immune and islet cells to address this question. First, as suggested by the reviewer, we have applied AMULET to publicly available snATAC-seq datasets that we could find, including data from Innate Lymphoid Cells (ILCs) in mice[3] (n=2) and CD45+ immune cells in mice[2] (n=2). We also down-sampled the PBMC samples at 5% increments starting at 50%. These provided us with more samples to systematically study the effect of the read coverage on AMULET’s performance in immune cells. Furthermore, to better estimate the effect in islets, we also applied AMULET on in-house islet snATAC-seq data from 13 more individuals (unpublished data). For both cell types, AMULET reached its peak performance when the median read count per nuclei was ~25K valid read pairs (~120K total reads per nucleus), suggesting that this is the sweet spot for our method (Response Figure 4). Interestingly, samples from human and mice followed the same trend. We observed differences between tissues as the reviewer expected, where AMULET was more effective in immune cells compared to islets (recall 90% versus 65%). As suggested by the reviewer, this could be due to the fact that immune and islet cells have different chromatin accessibility levels and patterns.*

*These new read depth analyses and results are included in the revised manuscript (revised Fig. 4) and we revised our manuscript (lines 187-207) to emphasize the read count recommendations for future users:*

*“AMULET’s performance improves with increased library depth. AMULET’s recall was better in highly-sequenced PBMC samples compared to islets (Fig. 3a,b). To further study the library depth effects on AMULET’s performance, we applied it on snATAC-seq data with different library depth: 1) publicly available mouse CD45+ cells[16] and Innate Lymphoid Cells (ILCs) data[17]; 2) in-house islet data from 13 more donors (raw data not provided); and 3) down-sampled PBMC1 and PBMC2 samples at 5% increments starting at 50%. To quantify AMULET’s performance, we simulated multiplets in each sample by randomly selecting nuclei pairs and used them to calculate recall. For immune cells (PBMCs, CD45+ cells, and ILCs), AMULET achieved 88-89% recall for samples when the median read count per nuclei was 25-28K valid read pairs, and we observed a linear trend between recall and read count per nuclei until a certain depth is achieved (Fig. 4d). Islets also exhibited a similar trend, where the relationship between AMULET’s recall and read depth followed a linear trend until a saturation point is achieved. Similar to immune cells, optimal performance was achieved around 25-29K valid read pairs per nuclei. Interestingly, the maximum recall achieved for islet samples was 52-66%. The variation in AMULET’s performance between immune and islet cells is likely due to the differences in their overall chromatin accessibility levels and patterns that might arise from distinct cellular differentiation potential and/or functions. We further investigated the number of valid read pairs required to detect multiplets using AMULET by randomly pairing 5% of the total nuclei per sample (repeated 100 times). We used AMULET to detect these artificial multiplets, then studied the read count distribution of the ones that are accurately identified. These analyses revealed that in both PBMCs and islets, ~25K valid read pairs per cell/nucleus is sufficient for detecting artificial multiplets with high recall using AMULET (Additional file 1: Figure S9). Together, these analyses show that AMULET’s performance depends on the read depth and that maximum performance is achieved when the median read count per nuclei is at least 25K valid read pairs regardless of the tissue type.”*

Reviewer 2: For the potential users, it would be helpful to report the computational needs, for example, CPU hours, RAM, run time on typical data set of different cell numbers, such as 5,000, 10,000, or even 100k.

*We thank Reviewer 2 for this suggestion. We measured the runtime and memory requirements for our method by studying its performance as a function of cell numbers and number of reads in 15 islet samples (Response Figure 5). These results showed that the runtime of AMULET scaled almost linearly with increased number of nuclei/valid reads, as expected based on the theoretical runtime complexity of O(nlogn) (n = number of reads) to sort the read positions. The amount of memory used in these runs did not exceed 3GB (Response Figure 4cd). These analyses suggest that for most samples, AMULET will generate results within hours using a standard laptop. We included these new analyses as a supplementary figure (revised Figure S11) and revised the discussion and methods regarding the runtime (lines 293-296):*

*“AMULET is a fast and efficient tool that can detect multiplets with a runtime that scales near linearly with the number of cells/valid reads (Additional file 1: Figure S11a,b), while requiring less than 3GB of memory (Additional file 1: Figure S11c,d). These analyses suggest that for most samples, AMULET will generate results within hours using a standard laptop.”*

**Response Figure 5.** Runtime and memory requirements for running AMULET for different cell numbers and read counts. Runtime scales linearly with respect to the number of (**a**) cells and (**b**) valid read pairs, in concordance with the theoretical expectations based on the worst-case runtime to sort the reads O(n*nlog(n)). AMULET requires less than 3GB of memory for up to 100k cells (**c**) and 2.5 billion valid read pairs (**d**).

*REFERENCES*

*1. Huang Y, McCarthy DJ, Stegle O. Vireo: Bayesian demultiplexing of pooled single-cell RNA-seq data without genotype reference. Genome Biol. 2019 Dec;20(1):273.*

*2. Warshauer JT, Belk JA, Chan AY, Wang J, Gupta AR, Shi Q, et al. A human mutation in STAT3 promotes type 1 diabetes through a defect in CD8+ T cell tolerance. Journal of Experimental Medicine. 2021 Aug 2;218(8):e20210759.*

*3. Bielecki P, Riesenfeld SJ, Hütter J-C, Torlai Triglia E, Kowalczyk MS, Ricardo-Gonzalez RR, et al. Skin-resident innate lymphoid cells converge on a pathogenic effector state. Nature [Internet]. 2021 Feb 3 [cited 2021 Jul 9]; Available from: http://www.nature.com/articles/s41586-021-03188-w*

**Second round of review**

**Reviewer 1**

My main concerns have been addressed.

However, there seems to be some discrepancy between the new Fig. 4d and the old Fig. 3c,d. In the new figure, the average recalls are close to 0.75 for median valid read pairs of ~15k (for PBMCs), whereas in the old figure, the average recall is between 0 and 0.2 for median valid read pairs between 10k and 21k. The recall for islet is also higher in the new figure. But the description of the method does not seem to indicate a big difference either in the algorithm or simulation. Could the authors explain the difference?

**Authors Response**

**Point-by-point responses to the reviewers’ comments:**

"My main concerns have been addressed. However, there seems to be some discrepancy between the new Fig. 4d and the old Fig. 3c,d. In the new figure, the average recalls are close to 0.75 for median valid read pairs of ~15k (for PBMCs), whereas in the old figure, the average recall is between 0 and 0.2 for median valid read pairs between 10k and 21k. The recall for islet is also higher in the new figure. But the description of the method does not seem to indicate a big difference either in the algorithm or simulation. Could the authors explain the difference?"

*We apologize for the confusion. In order to understand the effect of the read depth on our predictions, previously we were simulating multiplets by selecting nuclei pairs with different read depths (old Fig 3c,d). In these previous figures, the reported read depth is the sum of nuclei pairs (i.e., ~15K per pair, ~7.5K per nucleus). However, based on the reviewers’ feedback, we agreed that a better way to study this relation is by applying AMULET on more samples of different depths and on downsampled data (Figure 4d). In Figure 4d, the number of valid read pairs reported are the median read pair counts per nuclei in the entire sample (not pairs of nuclei). A fair comparison of these two approaches would be to compare ~30k median valid reads from old figure (since this is nuclei pairs) to the ~15K reads per nuclei in the new analyses, which both correspond to ~0.75 recall. We also want to clarify that these numbers will not exactly match since the first method focuses only on the analyses of the artificial doublets and the second one studies all nuclei in a given snATAC-seq sample. We are thankful to the reviewers for this great suggestion since these new analyses are more directly testing the effects of the read depth on the performance of AMULET. We therefore opted to replace the previous figure with the new one.*
